# Variations and expression features of CYP2D6 contribute to schizophrenia risk

**DOI:** 10.1038/s41380-020-0675-y

**Published:** 2020-02-11

**Authors:** Liang Ma, Anna Shcherbina, Sundari Chetty

**Affiliations:** 1 Department of Psychiatry and Behavioral Sciences, Stanford University School of Medicine, Stanford, CA 94305 USA; 2 Department of Biomedical Informatics, Stanford University, Stanford, CA 94305 USA; 3 Institute for Stem Cell Biology and Regenerative Medicine, Stanford University School of Medicine, Stanford, CA 94305 USA

**Keywords:** Psychiatric disorders, Genetics

## Abstract

Genome-wide association studies (GWAS) have successfully identified 145 loci implicated in schizophrenia (SCZ). However, the underlying mechanisms remain largely unknown. Here, we analyze 1497 RNA-seq data in combination with their genotype data and identify SNPs that are associated with expression throughout the genome by dissecting expression features to genes (eGene) and exon–exon junctions (eJunction). Then, we colocalize eGene and eJunction with SCZ GWAS using SMR and fine mapping. Multiple ChIP-seq data and DNA methylation data generated from brain were used for identifying the causal variants. Finally, we used a hypothesis-free (no SCZ risk loci considered) enrichment analysis to determine implicated pathways. We identified 171 genes and eight splicing junctions located within four genes (*SNX19*, *ARL6IP4*, *APOPT1*, and *CYP2D6*) that potentially contribute to SCZ susceptibility. Among the genes, *CYP2D6* is significantly associated with SCZ SNPs in eGene and eJunction. In-depth examination of the *CYP2D6* region revealed that a nonsynonymous single nucleotide variant rs16947 is strongly associated with a higher abundance of *CYP2D6* exon 3 skipping junctions. While we found rs133377 and other functional SNPs in high linkage disequilibrium with rs16947 (*r*^2^ = 0.9539), histone acetylation analysis showed they are located within active transcription start sites. Furthermore, our data-driven enrichment analysis showed that CYP2D6 is significantly involved in drug metabolism of codeine, tamoxifen, and citalopram. Our study facilitates an understanding of the genetic architecture of SCZ and provides new drug targets.

## Introduction

Schizophrenia (SCZ) is a debilitating, highly heritable, and polygenic psychiatric condition affecting roughly 1% of the population. While prior genome-wide association studies (GWASs) have yielded inconsistent findings [1] and candidate gene studies have identified risk SNPs and haplotypes [2], with increased sample size and improved study design, recent GWAS have successfully identified 145 risk loci associated with SCZ [3]. While these findings have identified regions in the genome harboring SCZ genes, almost all of the regions include multiple genes that are located within the same recombination hotspot intervals, making it challenging to identify causal genes.

One essential approach to determining the function of the identified SNPs is to combine GWASs with gene expression in postmortem human brains. Recently, several studies have applied these strategies to the 108 loci identified by the PGC in 2014 [4]. Using genotyped RNA-seq data generated by the CommonMind Consortium from postmortem dorsolateral prefrontal cortex (DLPFC), splicing QTLs were found to be significantly enriched in SCZ risk loci [5]. The enrichment of SCZ loci was also observed in another independent study of postmortem brain DLPFC samples [6], suggesting that combining these analytical techniques provides important insights into the mechanisms underlying SCZ.

Studies integrating eQTL and GWAS data have almost exclusively used quantified expression across multiple transcript features, including annotated genes as well as annotation-guided transcripts. Notably, junction calls from short RNA-seq reads are considerably more reliable than assembled transcripts [7]. However, only a limited number of studies employed exon–exon junctions to identify splicing transcripts that contribute to the underlying phenotypes [6, 8]. Recently, skipping of exon 2 and exon 3 of AS3MT was identified to contribute to SCZ risk using 495 postmortem brain cohorts [9]. Using the same specimens, we also successfully identified that the risk allele of SCZ SNPs is strongly associated with splicing junctions between exon 8 and exon 10 of *SNX19* [10].

Here, we leveraged genotype and brain expression data provided by the Genotype-Tissue Expression (GTEx) project to elucidate the functional properties and potential roles of eQTL and splicing expression features in the etiology of SCZ. Integrating the GTEx brain eQTLs with the most recent SCZ GWAS data [3], we evaluated specific effects of the identified SCZ risk variants on gene expression features in combination with epigenetics data, and examined how genes of expression features affect genetic networks. Splicing transcripts have long been predicted to play roles in disease development, but the role of splicing in SCZ remains poorly understood. We address this gap by systematically evaluating exon–exon junctions in the human brain. By colocalizing the results from our gene- and junction-level analyses, we identify the functional genomic variants and potential effective splicing transcripts underlying SCZ susceptibility.

## Materials and methods

An overview of our workflow can be found in Fig. S1.

### RNA-seq of postmortem brain

A total of 1497 human brain samples across 13 brain regions were used in this study (Tables 1 and S1). All of the postmortem brain samples were collected by the GTEx consortium. The sample procurement has been described previously [11]. Raw gene and exon–exon junction reads counts were retrieved from GTEx portal. Gene lengths were calculated using GENCODE v19 annotations [12]. We converted gene counts to RPKM values using the total number of aligned reads across the 22 autosomal chromosomes. Considering a median depth of 84 million reads in the sequencing (Table 1), we converted junction counts to RP80M values using the total number of aligned reads across the autosomal chromosomes, which can be interpreted as the number of reads supporting the junction in an average library size [6].

**Table 1:** Associations of the splicing junctions with SCZ risk SNPs across the 13 brain regions.

Tissue	*N*	Average mapped reads counts	ARL6IP4			APOPT1		CYP2D6	
			rs1790121			rs10431750		rs133377	
			Exon_2.3.1	Exon_2.3.2	Exon_1.4	Exon_2.4	Exon_3.4	Exon_3.4	Exon_2.4
Amygdala	88	83,912,256	1.45E–05	1.48E–10	0.0002	0.0165	0.021	0.0003	3.79E–06
ACC	109	88,136,513	3.16E–09	1.00E–13	2.39E–07	6.72E–05	2.40E–04	4.99E–08	5.34E–06
Caudate	144	86,167,404	4.26E–12	2.15E–26	1.01E–07	0.0046	9.52E–04	4.69E–11	8.47E–12
Cerebellar	125	89,274,842	1.38E–10	1.93E–25	0.0024	1.66E–04	0.0048	7.62E–13	9.09E–19
Cerebellum	154	82,622,299	5.23E–15	6.19E–30	0.0383	0.0092	7.67E–05	4.02E–10	1.47E–21
Cortex	136	82,272,249	1.05E–09	1.76E–26	0.0003	3.78E–05	2.76E–04	3.01E–07	5.20E–05
DLPFC	118	85,916,703	9.66E–15	2.85E–22	2.70E–05	3.86E–04	1.00E–04	2.65E–04	1.93E–06
Hippocampus	111	82,487,941	5.95E–09	2.64E–23	1.26E–06	0.0554	0.3127	3.02E–06	0.011
Hypothalamus	108	85,014,817	2.84E–11	3.45E–15	8.28E–04	0.011	5.07E–03	1.42E–08	6.22E–08
Nucleus	130	88,584,649	7.08E–10	6.76E–16	8.77E–11	0.0022	6.13E–05	2.38E–12	1.25E–13
Putamen	111	85,791,599	5.78E–05	4.97E–21	8.40E–04	0.8895	0.9736	3.72E–06	3.29E–06
Spinal	83	82,583,948	4.79E–08	4.11E–14	1.92E–04	0.0889	0.0568	0.0917	1.93E–04
Sub	80	80,973,047	0.0003	1.37E–14	0.0022	1	0.6795	3.35E–05	1.06E–04

False discovery rate (FDR) values are listed. Exon_8.10 in SNX19 was characterized in our prior work [10].

*ACC* anterior cingulate cortex (BA24), *Caudate* caudate (basal ganglia), *Cerebellar* cerebellar hemisphere, *DLPFC* dorsolateral prefrontal cortex (BA9), *Nucleus* nucleus accumbens (basal ganglia), *Putamen* putamen (basal ganglia), *Spinal* spinal cord (cervical c-1), *Sub* substantia nigra.

### Genotyping data

Whole-genome sequencing (WGS) datasets were retrieved from dbGap upon authentication by the GTEx Consortium (Accession: phs000424.v7.p2). We extracted a total of 42,585,769 genomic variants which were then filtered step-by-step by using PLINK 1.9 [13] if they: (1) had a genotype missing rate > 10% (272,734 variants); (2) had minor allele frequencies < 1% (31,110,395 variants); and (3) deviated from Hardy–Weinberg equilibrium (*p* value < 1E−5, 791,170 variants). Finally, we retained 10,411,470 variants for further analysis.

### *cis*-acting eQTL analysis

*cis*-eQTL association was implemented separately by feature type (gene and junction) using Matrix eQTL R package [14] with the additive linear model, treating log2-transformed expression levels of each measurement (RPKM and RP80M) as the outcome. Features without expression (average counts < 0) were excluded before eQTL analysis. To control for potential confounding factors, we adjust for ancestry (first three principle components (PCs) from the genotype data) [15], sex, and the first K PCs of the normalized expression features, where K was calculated separately by feature type using the sva Bioconductor package (gene: 13 PCs, junction: 13 PCs). False discovery rate (FDR) was assessed using the Benjamini–Hochberg algorithm (BH) across all *cis*-eQTL tests within each chromosome. We considered all variant–gene pairs (expression features to genes, eGene) and variant–junction pairs (eJunction) when the distance between features and SNP is <1MB.

### Colocalization of GWAS and eQTL associations

In order to assess the probability that molecular traits as estimated by *cis*-eQTLs and physiological traits as estimated by GWAS share the same causal variant, we colocalized 8,171,061 SCZ GWAS summary statistics [3] with our eGene and eJunction results. We used SMR and HEIDI tests for the colocalization analysis [16]. We used the default parameters and performed for the genes and junctions. SNPs with linkage disequilibrium (LD) *r*-squared between top-SNP > 0.90 or <0.05 were excluded as well as one of each pair of the remaining SNPs with LD r-squared >0.90. In addition, we conducted fine mappings of eGene and eJunction with SCZ GWAS separately. In the mapping process, any variants without either eQTL or GWAS association statistics were excluded.

### SNP annotation

ANNOVAR [17] was used for characterizing the categories of variants which include exonic, upstream, downstream, 3′-UTR, 5′-UTR, intronic, and intergenic regions. Roadmap/ENCODE2 chromatin-state signatures using a multivariate Hidden Markov Model from brain tissue and cell types were extracted and visualized using WashU Epigenome Browser. For the identification of the binding locations of transcription factors (TF), ENCODE TF binding data was downloaded [18]. Then, we used bedtools intersect [19] to match SNPs to ChIP-seq peaks. Brain histone ChIP-seq data were obtained from the ENCODE portal. In addition, histone acetylation QTLs data (H3K9Ac ChIP-seq) generated from DLPFC of 433 individuals and DNA methylation QTLs data generated from 468 individuals were obtained from Brain xQTL Server [20].

### Functional enrichment

We used three tools (WebGestalt [21], DAVID [22], and gProfiler [23]) for overrepresentation enrichment analysis which help us identify biological pathways that are significantly enriched in a gene list. The transcript features were mapped to Entrez Gene IDs and subsequently to KEGG pathway. Gene ontologies (GO) biological process and GO molecular function [24] were also calculated. FDR (BH) and fold enrichment were imputed. FDR < 0.001 was used as threshold.

## Results

### Transcriptome-wide association study

**Fig. 1:**
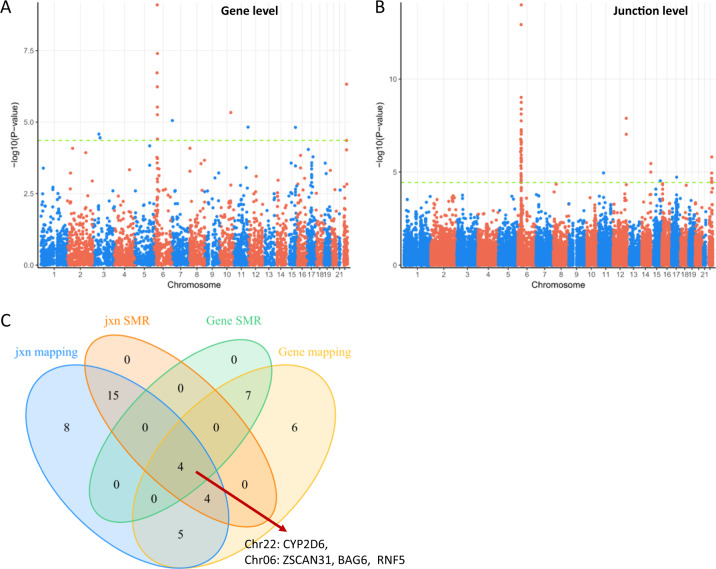
Identification of CYP2D6 as a top candidate for schizophrenia risk. Manhattan plot of DLPFC (Brodmann Area 9) in gene level (**a**) and junction level (**b**). **c** Venn diagram of significant eGene and eJunction in dorsolateral prefrontal cortex (BA9). CYP2D6 was observed in all overlapped combinations. Jxn, exon–exon junction. Manhattan plots and Venn diagrams of other 12 brain regions are shown in Supplementary Figs. S2, S3, and S8.

Alterations in the brain have been demonstrated to underlie cognitive deficits associated with SCZ, including impairments in working memory and cognitive flexibility [25]. To better understand the genetic interactions between expression features and genomic variations, we analyzed RNA-seq data of 13 brain regions from 1497 postmortem brains in combination with WGS data. We comprehensively identify *cis*-QTLs and expression features (gene and exon–exon splicing junctions) in the human brain with quality-controlled SNP genotyping data from the same individuals using Matrix eQTL [14] in a genome-wide manner (see Methods for details). To conservatively define eGene and eJunction SNPs, we applied BH procedure for multiple testing implemented in Matrix eQTL to the *p* values. To evaluate the extent of overlap between eQTL and GWAS signatures in SCZ and to identify putative causative genes from GWAS associations, we then performed colocalization analyses by SMR methods using default parameters. We also performed fine mapping for an in-depth investigation. After performing these procedures, we identified a total of 55 genes (SMR) and 89 genes (mapping) in eGene (Tables S2 and S3) and 186 junctions within 78 genes (SMR) and 343 junctions within 133 genes in eJunction (mapping) (Tables S4 and S5) across the 13 brain regions (Figs. 1, S2, and S3). Similar patterns of abundance of Cytochrome P450 2D6 (CYP2D6) gene Exon_2.4 and Exon_3.4 were observed across brain regions (Fig. S4) and developmental stages (Fig. S5), which is consistent with the functional connectivity between these brain regions [26].

As expected, most of the signals are from the major histocompatibility complex (MHC) region: 32/55 (SMR) and 51/89 (mapping) genes within eGene (Table S2 and Fig. 1a), and 41/78 and 74/133 (mapping) genes within eJunction (Tables S2–S5). The MHC is located in chromosome 6p21 and contains crucial regulators of the immune response. The MHC is the most gene dense and most polymorphic region of the human genome [27]. Complement component 4 (C4) structural variation was recently demonstrated to be related to the expression of C4A and C4B in postmortem brain [28]. In the current study, while C4A was detected in three brain regions in gene level by both SMR and fine mapping, C4A was also determined across ten brain regions in junction level by the two methods (Figs. 1b, c and S3 and Tables S4 and S5).

### Determination of splicing junctions

**Fig. 2:**
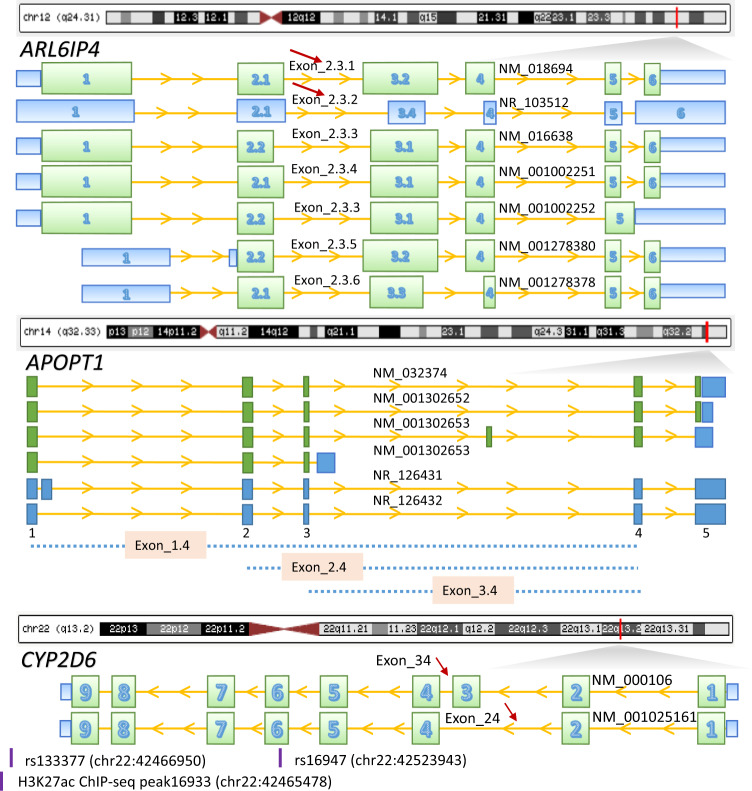
Splicing junctions on gene structures. Red bar at chromosomes indicates the gene physical position on the chromosome. Green boxes represent exons; blue boxes represent untranslated regions; solid yellow lines indicate introns; yellow arrows on yellow lines indicate gene transcriptional directions. Red arrows indicate identified junctions: Exon_2.3.1 (chr12:123465814–123466117) and Exon_2.3.2 (chr12:123465847–123466117) in *ARL6IP4*; Exon_1.4 (chr14:104029462–104053610), Exon_2.4 (chr14:104038158–104053610), and Exon_3.4 (chr14:104040508–104053610) in *APOPT1*; and Exon_3.4 (chr22:42524947–42525034) and Exon_2.4 (chr22:42522995–42523448) in *CYP2D6*. The association of SNX19 splicing junction Exon_8.10 has been previously characterized in our prior work [10]. Relative position of CYP2D6 SNPs and histone marker are indicated.

Alternative pre-mRNA splicing is a regulated process that results in a single gene coding for multiple proteins by including or excluding particular exons of a gene. This generates spliced mRNAs that direct the synthesis of a diverse set of proteins with varied biological functions. To illuminate our understanding of SCZ risk in splicing junction level, we systematically evaluated the exon–exon junctions in the human brain. We found eight splicing junctions located within four genes: Exon_2.3.1 and Exon_2.3.2 in *ARL6IP4*; Exon_1.4, Exon_2.4, and Exon_3.4 in *APOPT1*; Exon_3.4 and Exon_2.4 in *CYP2D6* (Fig. 2 and Table 1), including Exon 8.10 in *SNX19* as previously reported [10]. These junctions tag potential transcripts with alternative exonic boundaries (*ARL6IP4* Exon_2.3.1 and Exon_2.3.2) or exon skipping (*CYP2D6* Exon_3.4, *SNX19* Exon_8.10, and *APOPT1* Exon_1.4 and Exon_2.4) (Fig. 2). In prior work, we have systematically characterized splice junctions between exon 8 and exon 10 in *SNX19* [10].

### Effects of schizophrenia genetic risk on junctions

**Fig. 3:**
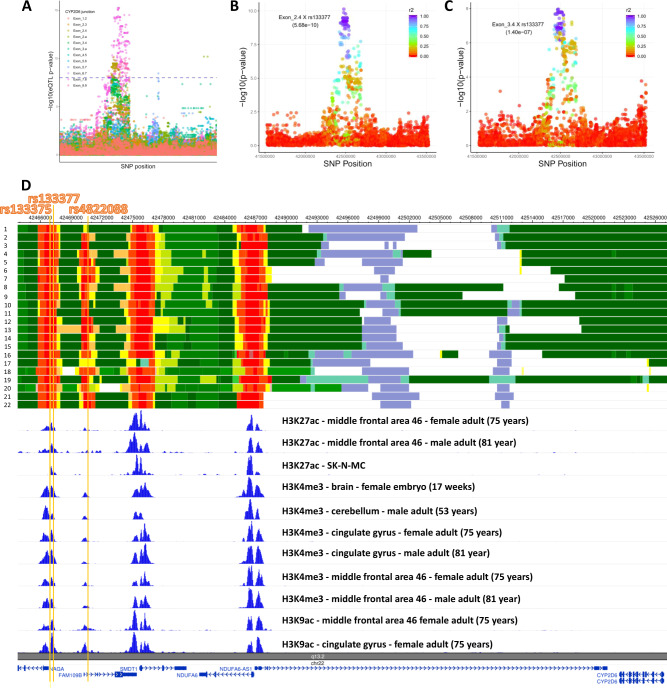
Identification of functional variants of CYP2D6. **a** Association of SNP-junction pairs (eJunction) of CYP2D6. Association of junctions Exon_2.4 (**b**) and Exon_3.4 (**c**) with SNPs upstream and downstream of rs133377. *r*^2^ was estimated using DLPFC samples. See association results from other 12 brain regions in Supplementary Figs. S10–S12. **d** Schizophrenia splicing SNPs are located within active transcription start sites. Upper section: ENCODE chromatin activation states in human brain from cortex-derived primary cultured neurospheres (1), angular gyrus (2, 3), anterior caudate (4, 5), cingulate gyrus (6, 7), germinal matrix (8), hippocampus middle (9, 10), inferior temporal lobe (11, 12), dorsolateral prefrontal cortex (13, 14), substantia nigra (15, 16), fetal brain male (17, 18), fetal brain female (19, 20), NH-A astrocytes primary cells (21, 22). See details in Supplementary Table S7. Middle section: ChIP-seq of H3K27ac, H3K4me3, and H3K9ac. Lower section: reference genes. Yellow bars represent coordinates of rs133375, rs133377, and rs4822088.

To determine how SCZ GWAS relates to feature expression, we next analyzed the trends of the associations. While SCZ risk allele is associated with down regulation of *APOPT1* junction Exon_3.4, all other risk alleles are associated with up regulation of junctions (Fig. S6). Interestingly, SCZ risk allele is associate with up regulation of *APOPT1* junction Exon_2.4 (Fig. 3). Opposing effects of SCZ risk allele on *APOPT1* Exon_3.4 and the other two junctions indicate their diverse functions. These robust effects are reproduced in the DLPFC and many other brain regions (Table 1). Based on the genomic recombination rate, the GWAS-eQTL loci are in a linkage block where recombination is not estimated to occur. In addition, a cluster of SNPs is in high LD with top SCZ GWAS-eQTL SNPs: *ARL6IP4* rs1790121, *APOPT1* rs10431750, and *CYP2D6* rs133377 (Fig. S7).

Strikingly, we found *CYP2D6* to be the core gene in 8 out of the 13 brain regions determined in both eGene and eJunction across the 13 brain regions using both SMR and fine mapping (Figs. 1, S2, S3, S8 and Tables S2–S5). Differential expression levels of CYP2D6 between SCZ and controls in neurons and oligodendrocytes isolated from postmortem brains were comparable across the two groups (Supplementary Fig. S9), suggesting that alternative splicing of the gene rather than total gene expression levels is associated with SCZ risk. Therefore, we further investigated the *CYP2D6* region. While multiple junctions were determined to be significant at the junction level across the 13 brain regions, we also found Exon_2.4, Exon_3.4, and Exon_7.8 to be significantly associated with SCZ risk SNPs (Figs. 3a–c, S10–S12 and Table S6). Interestingly, splicing occurred between exon 2 and exon 4 which resulted in maintaining or skipping exon 3. Exon_7.8 was included in all *CYP2D6* transcripts which represents gene level (Fig. 2).

### Functional characterization of eJunction SNPs in SCZ

Determining the underlying causal variants of complex disorders can be challenging because of the complex LD patterns between SNPs. We next attempted to functionally characterize eQTL SNPs by classifying the 255 SNPs in SCZ GWAS eJunction according to the definition in ANNOVAR [17]. As expected, most of the SNPs are nonexonic which accounts for 85.25% in SCZ GWAS eGene and 87.09% in SCZ GWAS eJunction (Fig. S13 and Table S6). There are a total of seven exonic single nucleotide variant (SNV), four of which are nonsynonymous. Only one SNV was found in our target genes, which is, rs16947, located within exon 6 of *CYP2D6* gene (NM_000106) (Fig. 2 and Table S6). Our CYP2D6 regional (2MB, about 5000 SNPs) LD analysis showed that SNPs in high LD with rs16947 are strongly associated with the abundance of splicing junctions (Exon_3.4 and Exon_2.4) in DLPFC and other brain regions (Table S6).

Enhancers have emerged as key *cis*-regulatory elements that play important regulatory roles in gene transcription, and they often reside distally from their target of regulation [29]. Using ENCODE chromatin activation states from brain, we found that SCZ splicing QTLs are located within active transcription start sites (Fig. 3d). Of the three SCZ splicing SNPs, rs133377 acts as an active transcription state across all the brains (Table S7). Three histone marks on H3K27ac, H3K4me3, and H3K9ac have been established to be associated with active transcription [30, 31]. As expected, the functional SNPs we identified are located within the chromatin modification peaks (Fig. 3d). Note that the functional SNPs are in high LD with rs16947 (*r*^2^ = 0.9539) (Fig. S14 and Table S6). In addition, we analyzed another independent H3K9ac ChIP-seq data and found rs133377 is significantly associated with a histone acetylation site, peak16933 in a 2 MB sliding window (chr22:42465478, *p* value = 3.78E−8) (Fig. S15 and Table S8). While Exon_2.4 and Exon_4.5 in CYP2D6 and Exon_7.8 in CYP2D7P1 were determined to be significantly associated with rs133377 in DLPFC, only Exon_2.4 is involved in splicing events. Using DNA methylation data of postmortem DLPFC brain from 468 individuals [20], we found CpG sites scattered around *CYP2D6* are significantly interacted with both functional SNPs (e.g., rs133377) and chromatin (e.g., peak16933) (Table S8), implying they function together to regulate CYP2D6 gene transcription. Additionally, we characterized the SNPs that are interacted with chromatin status and CpG sites, and find that the SNPs (e.g., rs133377) are overlaid with zinc finger motifs (e.g., ZNF263) and other TF binding sites (Table S9 and Fig. S16). Zinc finger motifs have known methyltransferase functions [32], implying the variants may influence gene expression by disrupting methyltransferase enzymes’ ability to recognize their motifs.

### Enrichment analysis of eJunction

**Fig. 4:**
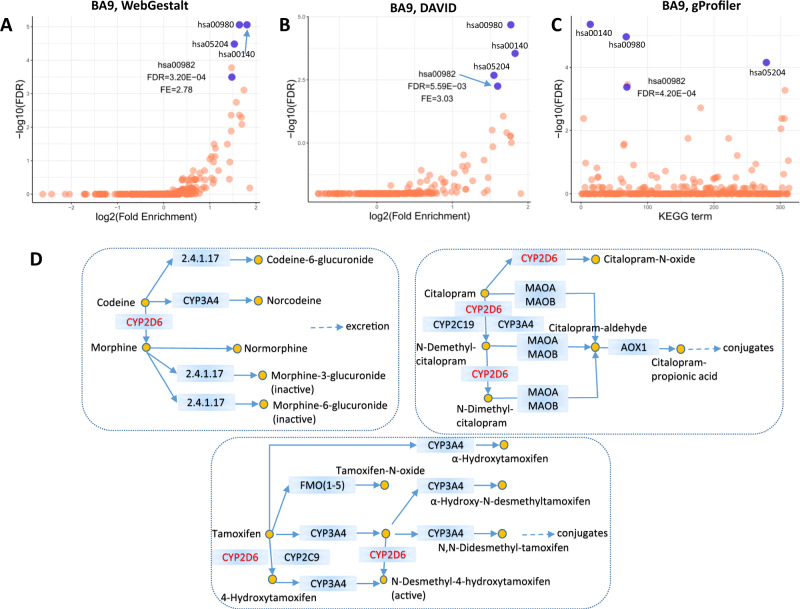
Pathway analysis of overlapped significant genes of eJunctions and eGenes. KEGG pathways enriched for 798 genes that are both significant in gene level and junction level in dorsolateral prefrontal cortex (BA9) by using WebGestalt (**a**), DAVID (**b**), and gProfiler (**c**). Blue dots are overlapped top KEGG pathways in BA9 using WebGestalt, DAVID, and gProfiler. FDR false discovery rate. Results of other 12 brain regions are shown in Supplementary Figs. S17 (WebGestalt), S18 (DAVID), and S19 (gProfiler). **d** CYP2D6 in the drug metabolism process of KEGG pathway hsa00982. 2.4.1.17: UGT1A(1,3–10), UGT2A(1–3), UGT2B(4,7,10,11,17,28).

To further verify our systematic genomics results and gain mechanistic insight, we next took an unbiased data-driven approach and performed a comprehensive gene-set pathway enrichment analysis to explore the potential functional implications of the genome-wide significant genes overlapped in junction level and gene level using WebGestalt, DAVID, and gProfiler across the 13 brain regions. An average of 822 genes from the 13 brain regions was determined and used for the enrichment analysis, and more than 300 pathways were imputed for each brain region by the three tools. Two top pathways that include CYP2D6 are associated with metabolism of xenobiotics by cytochrome P450 (hsa00980) and drug metabolism (hsa00982) and were consistently identified by each of the three tools across the 13 brain regions (Figs. 4a–c and S17–S19 and Tables S10–S12). When we took an in-depth examination of the KEGG hsa00982 pathway, we found that CYP2D6 was associated with the metabolic process of psychiatric drugs: tamoxifen, codeine, and citalopram (Figs. 4d and S17–S19). Similarly, GO biological process analyses showed *CYP2D6* and related genes play a role in steroid metabolic process in DLPFC and other brain regions (GO:0008202) (Table S13). GO molecular function analyses showed CYP2D6 and 13 related genes involved in drug binding (GO:0008144) in DLPFC and other brain regions (Table S14).

## Discussion

In this study, we analyzed a large-scale transcriptome derived from 1497 human postmortem brain samples in combination with WGS data. SCZ is a complex genetic disorder and over a hundred loci have been identified. However, it remains unclear how genetics affect the abundance of expression features. We identified potential effective splicing transcripts underlying SCZ susceptibility using the most recent GTEx brain RNA-seq data (released on June 30, 2017) [11] and 2018 SCZ GWAS summary statistics [3]. We identify *CYP2D6* to be an important candidate gene through several unbiased strategies: (1) by comparing gene overlapping using SMR and fine mapping in gene and junction level across 13 brain regions; and (2) through hypothesis-free overrepresentation enrichment analysis of eJunction genes. Furthermore, by analyzing multiple sources of ChIP-seq data generated from postmortem brains, we identify causal SNPs associated with CYP2D6.

Our principal findings include four major observations. First, we have identified a total of 171 genes across 13 brain regions for which genetic variation for expression colocalize with genetic variation for SCZ risk. Some differences in results are expected using alternative colocalization methods and references. Using 206 postmortem brain DLPFC samples from normal individuals, Takata et al. evaluated SCZ risk loci that are involved in splicing events based on assembled transcripts [5]. In the current study, we replicated reported genes, for example, *SNX19*, *ARL6IP4*, *APOPT1* [5], *C4A*, and *C4B* [28], and discovered new genes such as *CYP2D6*.

Second, we identified a list of exon–exon junctions that tag genetic transcripts. In prior work, most analyses focus almost exclusively on the marginal eQTL signal that typically represents the primary, or most significant, eQTL signal, rather than dissecting this signal into multiple independent features for each gene. While predicted transcripts are more likely to be used as a reference, we used exon–exon junctions to tag specific transcripts that considerably increase specificity. Our eJunction analyses identify eight splicing junctions that are located in four genes. Our recent study has systematically evaluated SNX19 Exon_8.10 transcripts and their potential susceptibility to SCZ [10]. APOPT1 was demonstrated to be located in mitochondria matrix, and involved in possessing an N-terminal mitochondrial targeting signal [33]. ARL6IP4 play a functional role in premRNA splicing [34, 35]. However, the mechanisms of APOPT1 and ARL6IP4 are still largely unknown.

Third, we illustrated an underlying mechanism for SCZ risk. There is accumulating evidence that CYP2D6 is involved in the metabolism of clinically used drugs [36]. A clinical study reported that CYP2D6 plays an important role in controlling the state of aripiprazole in the plasma which has been established as a form of treatment for SCZ [37]. CYP2D6 has also been reported to have significant effects on psychotic symptoms [38] and cognitive performance [39] of SCZ patients following risperidone treatment. Our data-driven enrichment analysis identified CYP2D6 as a key component of the drug metabolism pathways. CYP2D6/codeine has been highlighted as an antidepressant gene/drug pair in clinical therapy by Clinical Pharmacogenetics Implementation Consortium [40]. Codeine is bioactivated into morphine by CYP2D6 to exert its analgesic effect. Morphine, a strong opioid agonist, acts directly on the central nervous system and has been strongly implicated in addiction and SCZ pathophysiology [41]. Citalopram is a selective serotonin reuptake inhibitor used to treat major depression disorders [42]. Tamoxifen is well-known as an estrogen receptor modulator commonly used to treat breast cancer. In addition, we observed CYP2D6 to be involved in the metabolism of neuroactive steroids which are present in human postmortem brain tissue. In fact, concentrations of neuroactive steroids are known to be altered in subjects with SCZ and bipolar disorder [43]. Overall, these findings highlight CYP2D6 as an important candidate for further biological investigation.

Finally, by characterizing the properties of the detected eJunction SNPs, we found a nonsynonymous SNV, rs16947, to be associated with abundance of splicing junctions related to its exon 3 skipping. This leads to an in-frame deletion that shortens the translated protein by 51 amino acids. This risk allele of the SNV could result in an amino acid substitution that has been shown to reduce enzyme activity after recombinant cDNA transfection [44, 45]. On the other hand, we found three SNPs, which are in strong LD with rs16947, to be located within active chromatin modification regions in the brain, indicating that they are actively involved in direct regulation of CYP2D6 gene transcription [30, 31]. Thus, taken together, it is conceivable these alterations (changes in protein structure and/or chromatin modifications) affect the transcriptional activity of *CYP2D6*, leading to downstream changes in gene expression, which could further alter enzyme activity in drug metabolism. This alternative pre-mRNA splicing may also contribute to the extensive variability in CYP2D6 activity observed across individuals [46].

Although the sample size in this study is substantial (*N* = 1497), the sample size of each brain region is relatively small (mean = 115), so increasing sample size could help identify additional brain functional SNPs and splicing junctions with increased confidence. In addition, CRISPR genome editing of the causal SNPs, such as rs133377, on hiPSC-derived neurons would be of great value, although the technologies are still largely challenging [47].

In summary, we comprehensively analyzed expression features that are mediated by genomic markers across the human brain regions, described the characteristics of these SNPs, and demonstrated that the list of brain SNPs can be used to identify plausible candidate transcripts/variants that are causally associated with SCZ. These findings will be of great use in generating new animal and cellular models for SCZ.

## Supplementary information

Supplementary File

Supplementary Table S2

Supplementary Table S3

Supplementary Table S4

Supplementary Table S5

Supplementary Table S6

Supplementary Table S7

Supplementary Table S8

Supplementary Table S9

Supplementary Table S10

Supplementary Table S11

Supplementary Table S12

Supplementary Table S13

Supplementary Table S14

## Data Availability

PLINK 1.9, https://www.cog-genomics.org/plink/. Matrix eQTL R package, http://www.bios.unc.edu/research/genomic_software/Matrix_eQTL/. sva Bioconductor package, https://bioconductor.org/packages/release/bioc/html/sva.html. SMR, https://cnsgenomics.com/software/smr/#Overview. ANNOVAR, http://annovar.openbioinformatics.org/en/latest/. WashU Epigenome Browser, https://epgg-test.wustl.edu/browser/. GTEx portal, https://gtexportal.org/home/datasets.
