# Supplementary material for: Variations and expression features of CYP2D6 contribute to schizophrenia risk
Source: Mol Psychiatry. 2020 Feb 11;26(6):2605–15. doi: 10.1038/s41380-020-0675-y (PMC8440189; doi:10.1038/s41380-020-0675-y)
Supplement: Supplementary file 1 — Supplementary File [file 41380_2020_675_MOESM1_ESM.pdf]

Supplementary file

Variations and expression features of CYP2D6 contribute to schizophrenia risk

Liang Ma[1*], Ph.D., Anna Shcherbina[2], Sundari Chetty[1,3*], Ph.D.

1. Department of Psychiatry and Behavioral Sciences, Stanford University School of Medicine, Stanford, California 94305, USA

2. Department of Biomedical Informatics, Stanford University, Stanford, California 94305 USA

3. Institute for Stem Cell Biology and Regenerative Medicine, Stanford University School of Medicine, Stanford, California 94305, USA

*Correspondence to: liangma1@stanford.edu; chettys@stanford.edu

Supplementary Materials and methods

An overview of our workflow can be found in Figure S1.

RNA-seq of postmortem brain

A total of 1,497 human brain samples across 13 brain regions were used in this study (Table 1). All of the postmortem brain samples were collected by the GTEx consortium. The sample procurement has been described previously (GTEx_Consortium, et al., 2017). Raw gene and exon-exon junction reads counts were retrieved from GTEx portal (https://gtexportal.org/home/datasets). Gene lengths were calculated using GENCODE v19 annotations (Harrow, et al., 2012). We converted gene counts to RPKM (Reads per Kilobase per Million mapped reads) values using the total number of aligned reads across the 22 autosomal chromosomes. Considering a median depth of 84 million reads in the sequencing (Table 1), we converted junction counts to RP80M values (reads per 80 million mapped) using the total number of aligned reads across the autosomal chromosomes, which can be interpreted as the number of reads supporting the junction in an average library size (Jaffe, et al., 2018). RNA-seq data of CYP2D6 was obtained from the BrainSpan atlas (https://www.brainspan.org/static/home) to assess CYP2D6 expression across developmental stages. RNA-seq data in neurons (27 SCZ and 23 controls) and oligodendrocytes (23 SCZ and 18 controls) isolated from postmortem brains were collected from Mendizabal et al (Mendizabal, et al., 2019), and we performed Wilcoxon rank-sum tests to check for differential expression.

Genotyping data

Whole-genome sequencing (WGS) datasets were retrieved from dbGap upon authentication by the GTEx Consortium (Accession: phs000424.v7.p2). Genomic variants were called using GATK HaplotypeCaller (GTEx_Consortium, et al., 2017). We extracted a total of 42,585,769 genomic variants which were then filtered step-by-step by using PLINK 1.9 (Chang, et al., 2015) if they: 1) had a genotype missing rate >10% (272,734 variants); 2) had minor allele frequencies < 1% (31,110,395 variants); and 3) deviated from Hardy–Weinberg equilibrium (p-value < 1E−5, 791,170 variants). Finally, we retained 10,411,470 variants for further analysis.

cis-acting eQTL analysis

cis-eQTL association was implemented separately by feature type (gene and junction) using Matrix eQTL R package (Shabalin, 2012) with the additive linear model, treating log2-transformed expression levels of each measurement (RPKM and RP80M) as the outcome. Features without expression (average counts < 0) were excluded before eQTL analysis. To control for potential confounding factors, we adjust for ancestry (first three principle components (PCs) from the genotype data) (Price, et al., 2006), sex, and the first K PCs of the normalized expression features, where K was calculated separately by feature type using the sva Bioconductor package (gene: 13 PCs, junction: 13 PCs). False discovery rate (FDR) was assessed using the Benjamini-Hochberg algorithm (BH) across all cis-eQTL tests within each chromosome. We considered all variant-gene pairs (eGene) and variant-junction pairs (eJunction) when the distance between features and SNP is less than 1MB.

Co-localization of GWAS and eQTL associations

In order to assess the probability that molecular traits as estimated by cis-eQTLs and physiological traits as estimated by GWAS share the same causal variant, we co-localized 8,171,061 SCZ GWAS summary statistics (Pardinas, et al., 2018) with our eGene and eJunction results. We used SMR and HEIDI tests for the co-localization analysis (Zhu, et al., 2016). We used the default parameters and performed for the genes and junctions. SNPs with LD r-squared between top-SNP > 0.90 or < 0.05 were excluded as well as one of each pair of the remaining SNPs with LD r-squared > 0.90. In addition, we conducted fine

mappings of eGene and eJunction with SCZ GWAS separately. In the mapping process, any variants without either eQTL or GWAS association statistics were excluded.

Functional variant annotation

ANNOVAR (Wang, et al., 2010) was used for characterizing the categories of variants which include exonic, upstream, downstream, 3'-UTR, 5'-UTR, intronic, and intergenic regions. Roadmap/ENCODE2 chromatin-state signatures using a multivariate Hidden Markov Model (chromHMM) from brain tissue and cell types were extracted and visualized using WashU Epigenome Browser (http://epigenomegateway.wustl.edu/legac/) (Supplementary Table S7). For the identification of the binding locations of transcription factors (TF), ENCODE TF binding data was downloaded (Kheradpour and Kellis, 2014). Then, we used BEDTools intersect (Quinlan and Hall, 2010) to match SNPs to ChIP-seq peaks.

Chromatin accessibility analysis was performed to check whether CYP2D6-associated variants disrupted known transcription factor (TF) binding sites for TF's active in the brain. Variants were overlapped with ChromHMM (Ernst and Kellis, 2012) annotations using the ChromHMM 15-state model in available brain samples from the Roadmap Project (https://egg2.wustl.edu/roadmap/web_portal/): E071- Brain Hippocampus Middle, E074- Brain Substantia Nigra, E068-Brain Anterior Caudate, E069- Brain Cingulate Gyrus, E072 – Brain Inferior Temporal Lobe, E067 – Brain Angular Gyrus, E073 – Brain Dorsolateral Prefrontal Cortex, E070 – Brain Germinal Matrix, E082 – Fetal Brain Female, E081 – Fetal Brain Male. Variants that overlapped with active TSS or enhancer states in one or more of these tissues were flagged as potential disruptors of transcriptional regulation for further follow up.

Variants were also checked for overlap with DNAse peaks in E081 and E082, as well as H3K27ac peaks in E071, E074, E068, E069, E072, E067, E073. If a variant in an enhancer of active TSS region from the above analysis also overlapped a DNAse or H3K27ac peak, it was flagged for further follow up.

Variants that passed the above two filters were overlapped with known transcription factor binding motif sites in the hg19 genome. Known motif sites were found by running the FIMO algorithm (Grant, et al., 2011) version 5.1 on the CIS-BP (Weirauch, et al., 2014) single species DNA dataset included with the MEME suite (http://meme-suite.org/db/motifs).

Variants were overlapped with the CIS-BP known motif sites and a check was performed whether the SNP overlapped a base pair with a non-negligible position frequency matrix (PFM) score – thresholds for whether the base was significant in the PFM were PFM-specific and determined by visual inspection of the PFM in TomTom (Gupta, et al., 2007).

In an additional analysis, brain histone ChIP-seq data were obtained from the ENCODE portal (https://www.encodeproject.org/). H3K27ac ChIP-seq data were generated from middle frontal area 46 of an adult female (75 years) (ENCFF828WUR), middle frontal area 46 of an adult male (81 years) (ENCFF437REF), and SK-N-MC (ENCFF874TEV). H3K4me3 ChIP-seq data were generated from the brain of a female embryo (17 weeks) (ENCFF780JKM), cerebellum of an adult male (53 years) (ENCFF343VSH), cingulate gyrus of an adult female (75 years) (ENCFF643RUU), cingulate gyrus of an adult male (81 years) (ENCFF067IEM), middle frontal area 46 of an adult female (75 years) (ENCFF488MLR), and middle frontal area 46 of an adult male (81 years) (ENCFF440XBZ). H3K9ac ChIP-seq data were generated from the middle frontal area 46 of an adult female (75 years) (ENCFF576NUZ) and cingulate gyrus of an adult female (75 years) (ENCFF764DDD). Peaks from these additional histone marks were also intersected with variants to check for disruption of active enhancer regions.

In addition, histone acetylation QTLs data (H3K9Ac ChIP-seq) generated from DLPFC of 433 individuals and DNA methylation QTLs data generated from 468 individuals were obtained from Brain xQTL Server (Ng, et al., 2017).

Functional enrichment

We used three tools (WebGestalt (Wang, et al., 2017), DAVID (Huang da, et al., 2009), and gProfiler (Reimand, et al., 2011)) for overrepresentation enrichment analysis which help us identify biological pathways that are significantly enriched in a gene list. The transcript features were mapped to Entrez Gene IDs and subsequently to KEGG pathway. Gene ontologies (GO) biological process and GO molecular function (Gene_Ontology_Consortium, 2015) were also calculated. FDR (BH) and fold enrichment were imputed. FDR < 0.001 was used as threshold.

Code availability

PLINK 1.9, https://www.cog-genomics.org/plink/. Matrix eQTL R package, http://www.bios.unc.edu/research/genomic_software/Matrix_eQTL/. sva Bioconductor package, https://bioconductor.org/packages/release/bioc/html/sva.html. SMR, https://cnsgenomics.com/software/smr/#Overview. ANNOVAR, http://annovar.openbioinformatics.org/en/latest/. WashU Epigenome Browser, https://epgg-test.wustl.edu/browser/. GTEx portal, https://gtexportal.org/home/datasets. BrainSpan atlas, https://www.brainspan.org/static/home. Roadmap Project, https://egg2.wustl.edu/roadmap/web_portal/. MEME suite, http://meme-suite.org/db/motifs. ENCODE portal, https://www.encodeproject.org/.

Legend

Legend of Supplementary Table

Supplementary Table S1. Demographic information

Supplementary Table S2. SNPs that are significant in schizophrenia GWAS are significantly associated with genes in 13 brain regions by SMR & HEIDI methods

Supplementary Table S3. SNPs that are significant in schizophrenia GWAS are significantly associated with genes in 13 brain regions by mapping

Supplementary Table S4. SNPs that are significant in schizophrenia GWAS are significantly associated with junctions in 13 brain regions by SMR & HEIDI methods

Supplementary Table S5. SNPs that are significant in schizophrenia GWAS are significantly associated with junctions in 13 brain regions by mapping

Supplementary Table S6. Schizophrenia risk SNPs are significantly associated with CYP2D6 junctions across 13 brain regions

Supplementary Table S7. Samples and chromatin state of CYP2D6 SNPs shown in Figure 3D

Supplementary Table S8. Association of CYP2D6 schizophrenia risk eJunction SNPs with histone acetylation peaks and CpG sites

Supplementary Table S9. Functional characterization of schizophrenia risk SNPs around CYP2D6

Supplementary Table S10. KEGG pathway of overlapped genes regulated by eGene and eJunction SNPs across the 13 brain regions by WebGestalt

Supplementary Table S11. KEGG pathway of overlapped genes regulated by eGene and eJunction SNPs across the 13 brain regions by DAVID

Supplementary Table S12. KEGG pathway of overlapped genes regulated by eGene and eJunction SNPs across the 13 brain regions by gProfiler

Supplementary Table S13. Steroid metabolic process of gene ontologies (GO:0008202) across multiple brain regions by DAVID

Supplementary Table S14. Drug binding of gene ontologies (GO:0008144) across multiple brain regions by DAVID

Legend of Supplementary Figure

Supplementary Figure S1. Flowchart for splicing transcript & variations identification.

Supplementary Figure S2. Manhattan plot of the 12 brain regions in gene level. Manhattan plots of dorsolateral prefrontal cortex (BA9) are shown in Figure 1.

Supplementary Figure S3. Manhattan plot of the 12 brain regions in junction level. Manhattan plots of dorsolateral prefrontal cortex (BA9) are shown in Figure 1.

Supplementary Figure S4. Abundance of CYP2D6 (A), Exon_2.4 (B) and Exon_3.4 (C) across the 13 brain regions. Distribution of colors denotes different brain regions.

Supplementary Figure S5. Expression pattern of CYP2D6 across developmental stages.

Supplementary Figure S6. Schizophrenia genetic risk effect on exon-exon junctions. Association of SNP-junction pairs: rs1790121 - ARL6IP4 Exon_2.3.1 (up) and Exon_2.3.2 (up); rs10431750 - APOPT1 Exon_1.4 (up) and Exon_2.4 (up) and Exon_3.4 (down); rs133377 - CYP2D6 Exon_34 (up) and Exon_24 (up). The junction abundance is from DLPFC. Red arrow indicates schizophrenia risk allele is associated with up regulation of junction, while blue arrow is associated with downregulation. SNX19 splicing junction Exon_8.10 was shown in our recent paper (Ma, et al., 2019).

Supplementary Figure S7. Regional schizophrenia GWAS signature co-localized with our identified variants that influence splicing of APOPT1 (Exon_1.4 and Exon_2.4 and Exon_3.4), CYP2D6 (Exon_34 and Exon_24). Linkage disequilibrium is colored with respect to ARL6IP4 rs1790121, APOPT1 rs10431750, and CYP2D6 rs133377. See SNX19 in our prior work (Ma, et al., 2019).

Supplementary Figure S8. Venn diagram of eGene and eJunction analysis across the 12 brain regions. Manhattan plots of dorsolateral prefrontal cortex (BA9) are shown in Figure 1.

Supplementary Figure S9. CYP2D6 gene expression levels in schizophrenia (n=41) and control (n=50) oligodendrocytes and neurons (Mendizabal, et al., 2019). The Wilcoxon rank sum test (2-sided, expression in controls greater than expression in schizophrenia, expression in schizophrenia greater than controls) was performed in oligodendrocytes (A), neurons (B), and the combined sample set of oligodendrocytes and neurons (C).

Supplementary Figure S10. Association of SNP-junction pairs (eJunction) of CYP2D6. See association results from DLPFC regions in Figure 4A.

Supplementary Figure S11. Association of junctions Exon_2.4 with SNPs upstream and downstream of rs133377. r2 was estimated using corresponding brain regions. See association results from DLPFC regions in Figure 4B.

Supplementary Figure S12. Association of junctions Exon_3.4 with SNPs upstream and downstream of rs133377. r2 was estimated using corresponding brain regions. See association results from DLPFC regions in Figure 4C.

Supplementary Figure S13. Characterization of identified schizophrenia GWAS eJunction SNPs. Pie charts indicating proportions of SNPs annotated with each functional category (exonic, upstream, downstream, 3’-UTR, 5’-UTR, splicing, intronic and intergenic).

Supplementary Figure S14. Linkage disequilibrium (LD) plot of identified schizophrenia GWAS functional SNPs. r2 was estimated using DLPFC data. See LD r2 at Supplementary Table S6.

Supplementary Figure S15. Association of rs133377 (chr22:42466950) with 70 histone acetylation peaks in 2 MB region around CYP2D6.

Supplementary Figure S16: Functional annotation for rs133377 (A) and rs4822088 (B). SNPs were overlapped with ChromHMM (15-state map) in Roadmap brain regions, DNAse data in Roadmap fetal brain samples (E081, E082 datasets), and H3K27ac Roadmap ChIP-seq datasets in the brain. Known TF binding sites in hg19 from FIMO and CISBP databases are overlapped with the SNP positions in the insets.

Supplementary Figure S17. Pathway analysis of overlapped significant genes of eJunctions and eGenes by WebGestalt across 12 brain regions.

Supplementary Figure S18. Pathway analysis of overlapped significant genes of eJunctions and eGenes by DAVID across 12 brain regions.

Supplementary Figure S19. Pathway analysis of overlapped significant genes of eJunctions and eGenes by gProfiler across 12 brain regions.

Supplementary Table

Supplementary Table S1. Demographic information

Tissue	Sample size	Sex		Race					Age		RIN		Mapped reads	
		M	F	1	2	3	4	99	Mean	SD	Mean	SD	Mean	SD
Amygdala	88	57	31	0	7	81	0	0	57.19	11.30	6.74	0.63	83,912,256	23,252,095
Anterior cingulate cortex (BA24)	109	73	36	0	6	102	0	1	58.17	10.63	6.87	0.79	88,136,513	28,238,187
Caudate (basal ganglia)	144	101	43	1	15	126	1	1	57.78	10.42	7.66	0.81	86,167,404	26,285,973
Cerebellar Hemisphere	125	89	36	1	10	113	0	1	57.11	11.45	7.80	0.97	89,274,842	24,376,773
Cerebellum	154	105	49	1	14	138	0	1	57.09	10.80	6.85	0.67	82,622,299	19,956,754
Cortex	136	90	46	1	15	120	0	0	57.28	10.62	6.59	0.62	82,272,249	19,082,750
Frontal Cortex (BA9)	118	83	35	0	14	104	0	0	58.14	9.92	7.36	0.85	85,916,703	25,737,545
Hippocampus	111	73	38	0	11	99	0	1	57.52	11.32	6.97	0.77	82,487,941	21,514,440
Hypothalamus	108	76	32	1	9	98	0	0	57.82	10.98	7.07	0.75	85,014,817	40,962,263
Nucleus accumbens (basal ganglia)	130	88	42	1	13	114	1	1	57.35	11.00	7.32	0.90	88,584,649	24,201,838
Putamen (basal ganglia)	111	79	32	1	11	98	0	1	57.34	10.46	7.30	0.89	85,791,599	26,257,802
Spinal cord (cervical c-1)	83	49	34	1	6	76	0	0	57.45	11.10	7.33	0.73	82,583,948	22,689,374
Substantia nigra	80	52	28	1	9	70	0	0	57.55	11.15	6.78	0.74	80,973,047	20,390,526

Sex: M=Male; F=Female

Race: 1=Asian; 2=Black or African American; 3=Caucasian; 4=American Indian or Alaska Native; 99=Unknown

Supplementary Figure

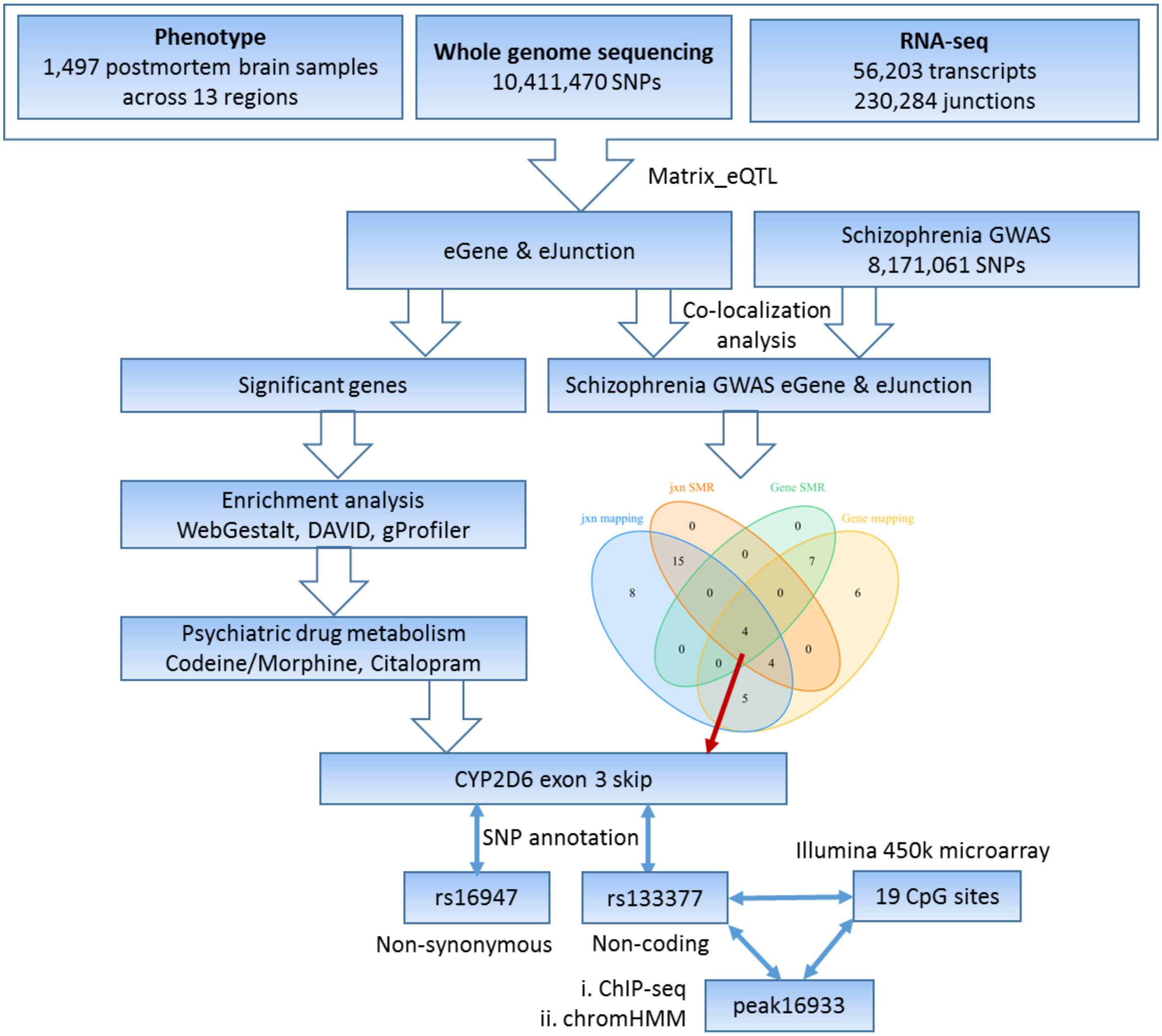

Supplementary Figure S1. Flowchart for splicing transcript & variations identification.
eGene, variant-gene pairs; eJunction, variant-gene pairs

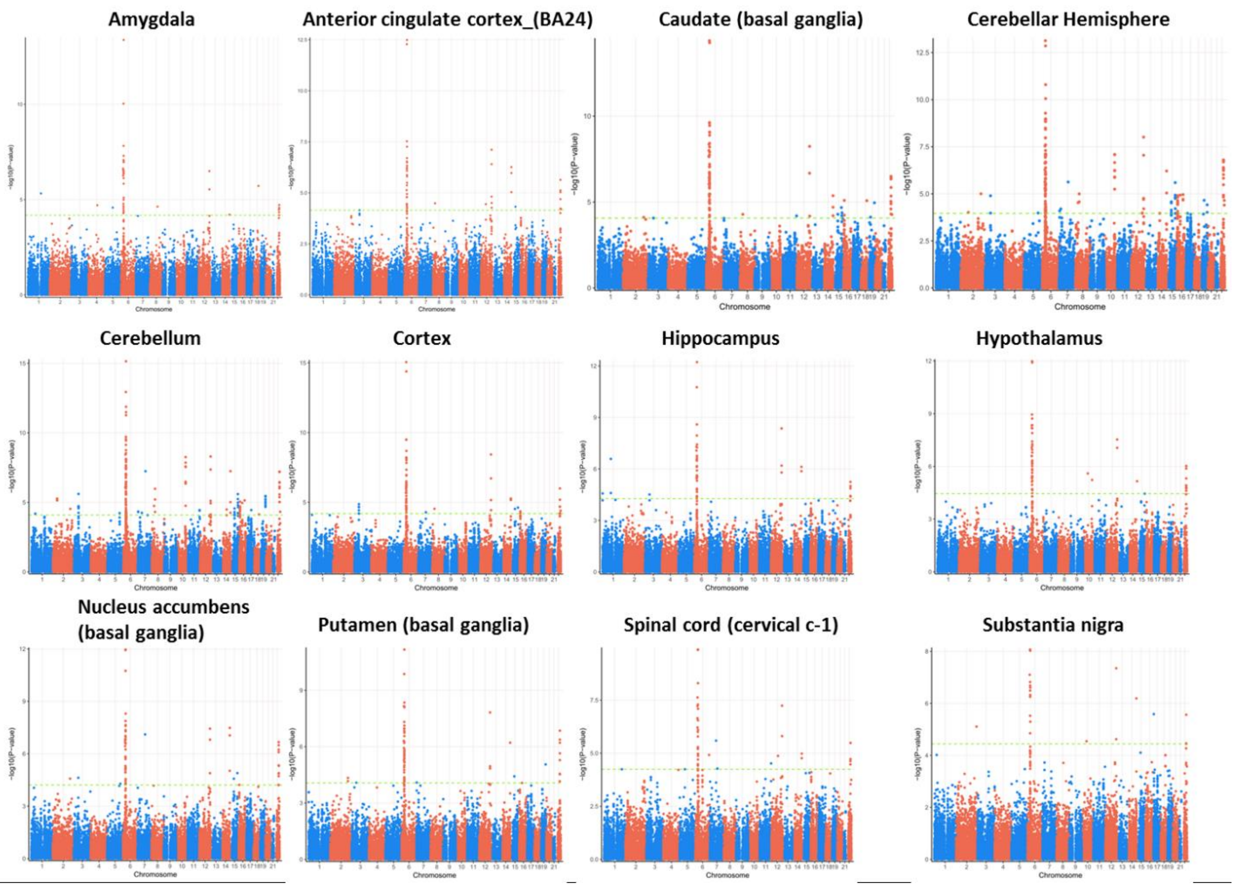

Supplementary Figure S2. Manhattan plot of the 12 brain regions in gene level. Manhattan plots of dorsolateral prefrontal cortex (BA9) are shown in Figure 1.

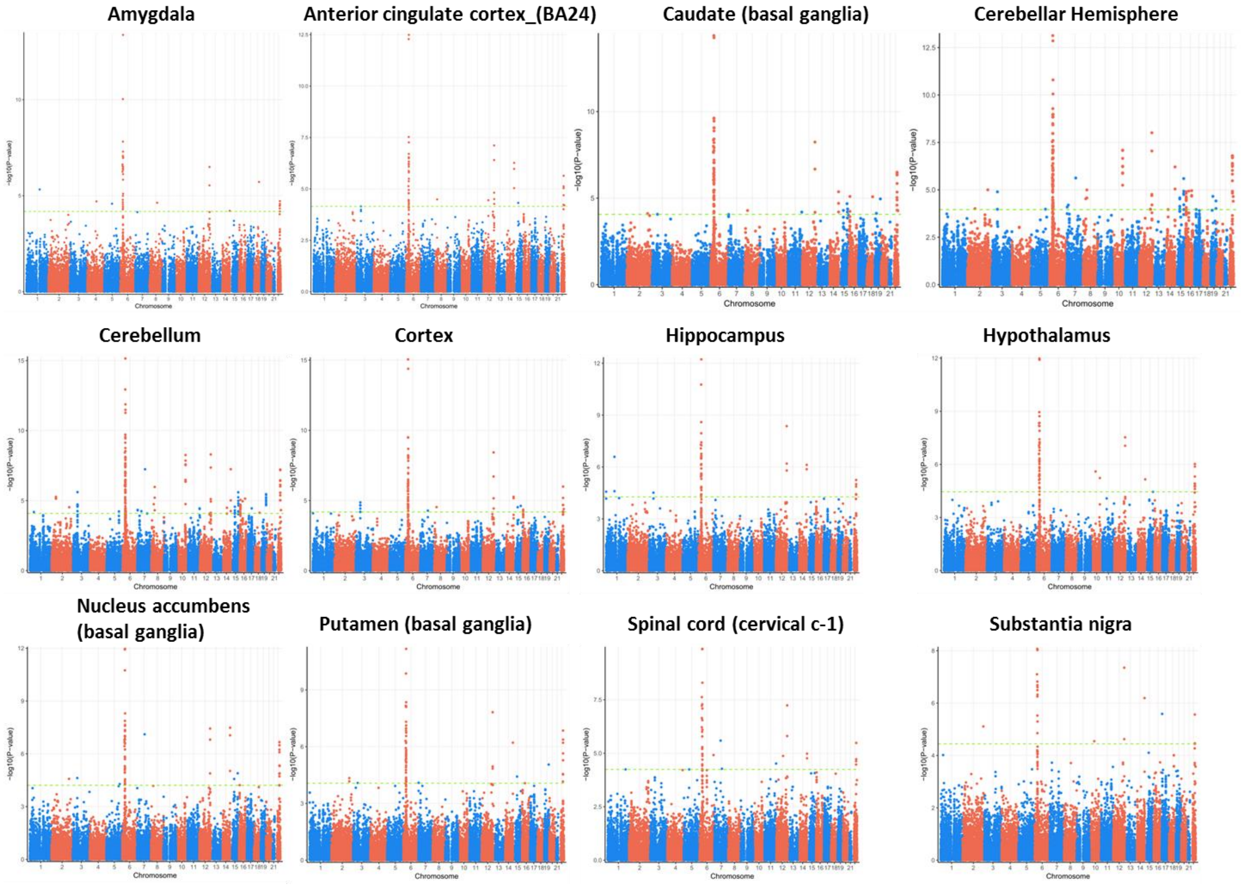

Supplementary Figure S3. Manhattan plot of the 12 brain regions in junction level. Manhattan plots of dorsolateral prefrontal cortex (BA9) are shown in Figure 1.

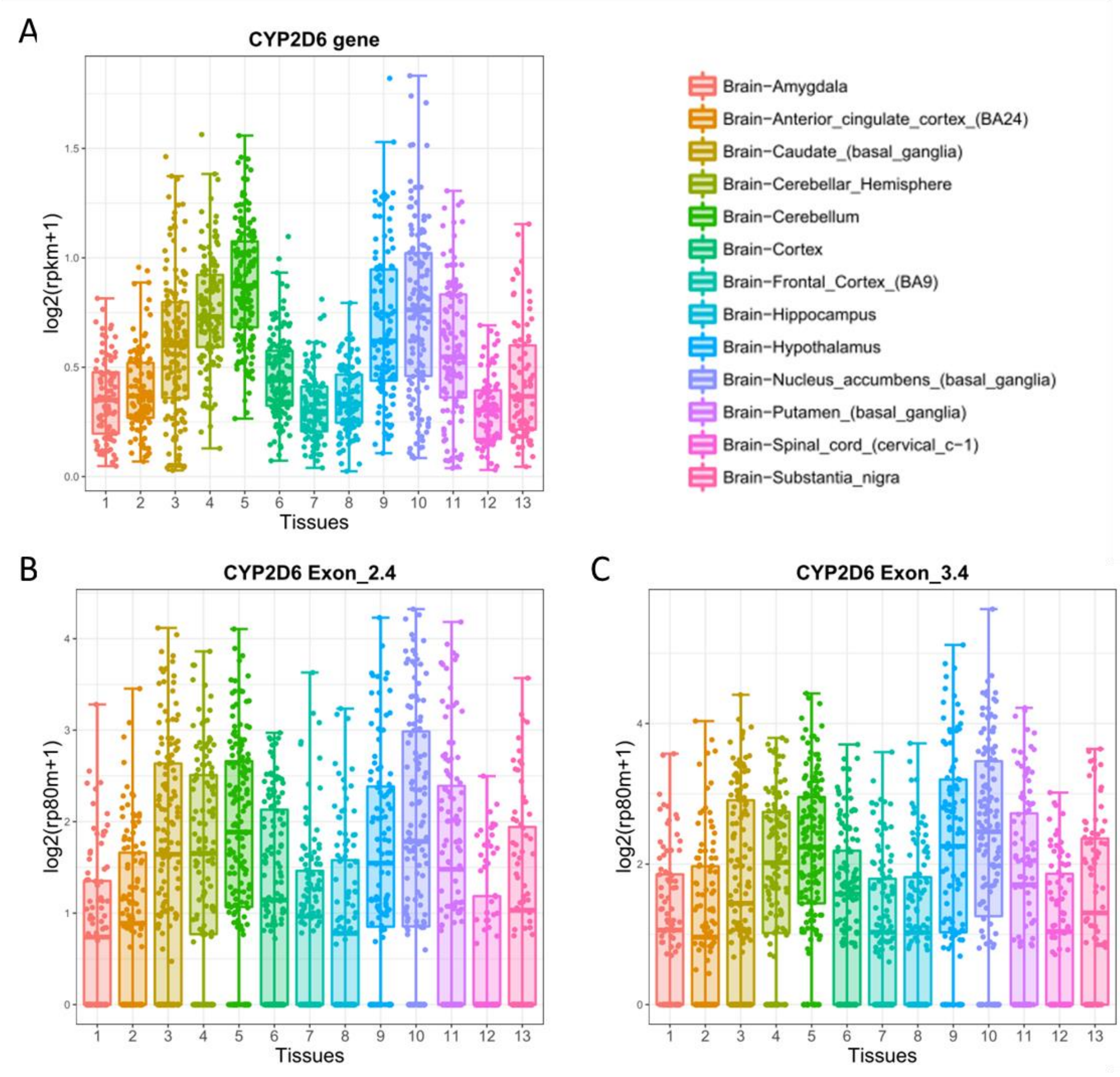

Supplementary Figure S4. Abundance of CYP2D6 (A), Exon_2.4 (B) and Exon_3.4 (C) across the 13 brain regions. Distribution of colors denotes different brain regions.

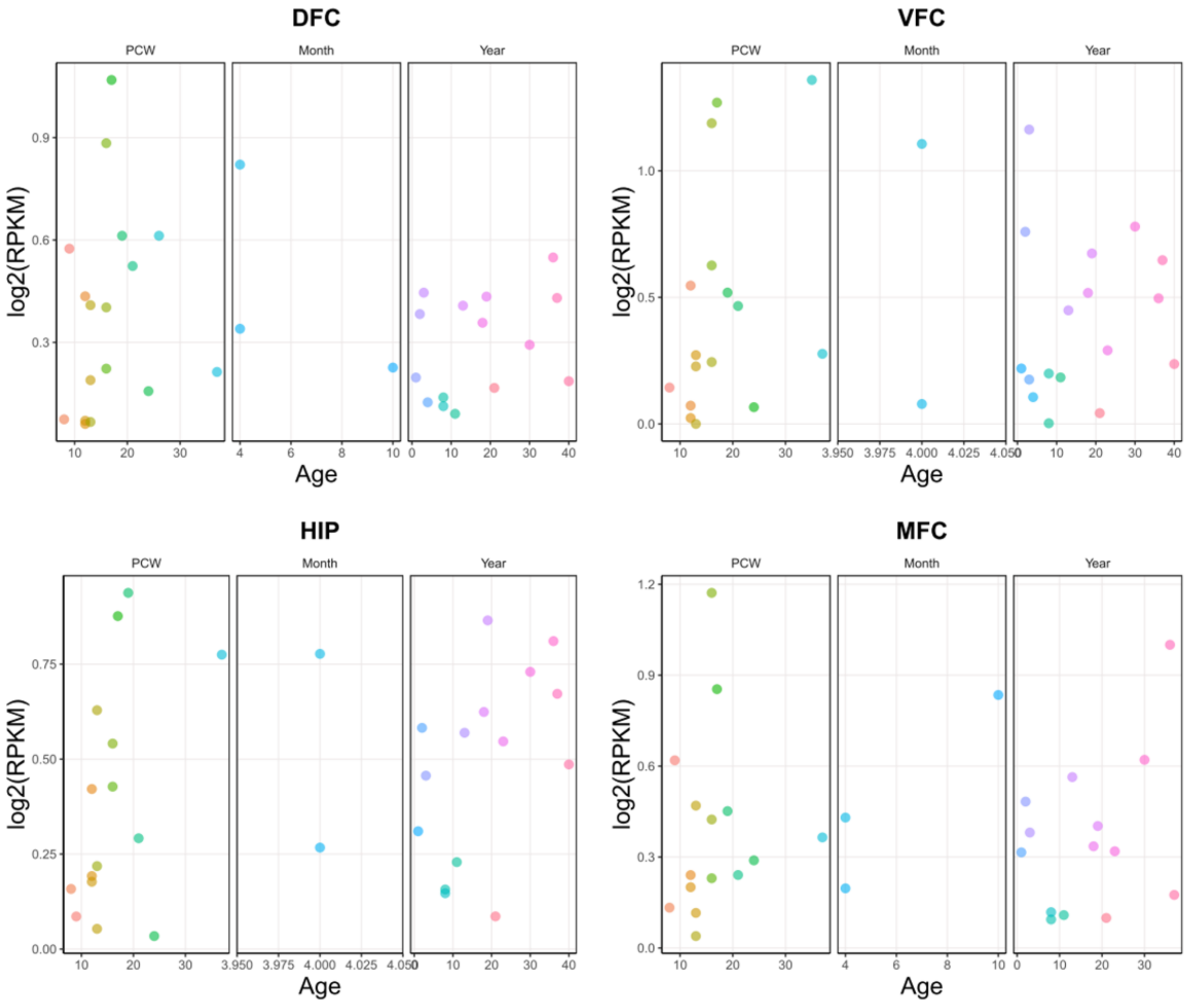

Supplementary Figure S5. Expression pattern of CYP2D6 across developmental stages.

DFC, dorsolateral prefrontal cortex; VFC, ventrolateral prefrontal cortex; HIP, hippocampus; MFC, anterior cingulate cortex; PCW, postconceptional weeks. Colors correspond to different samples.

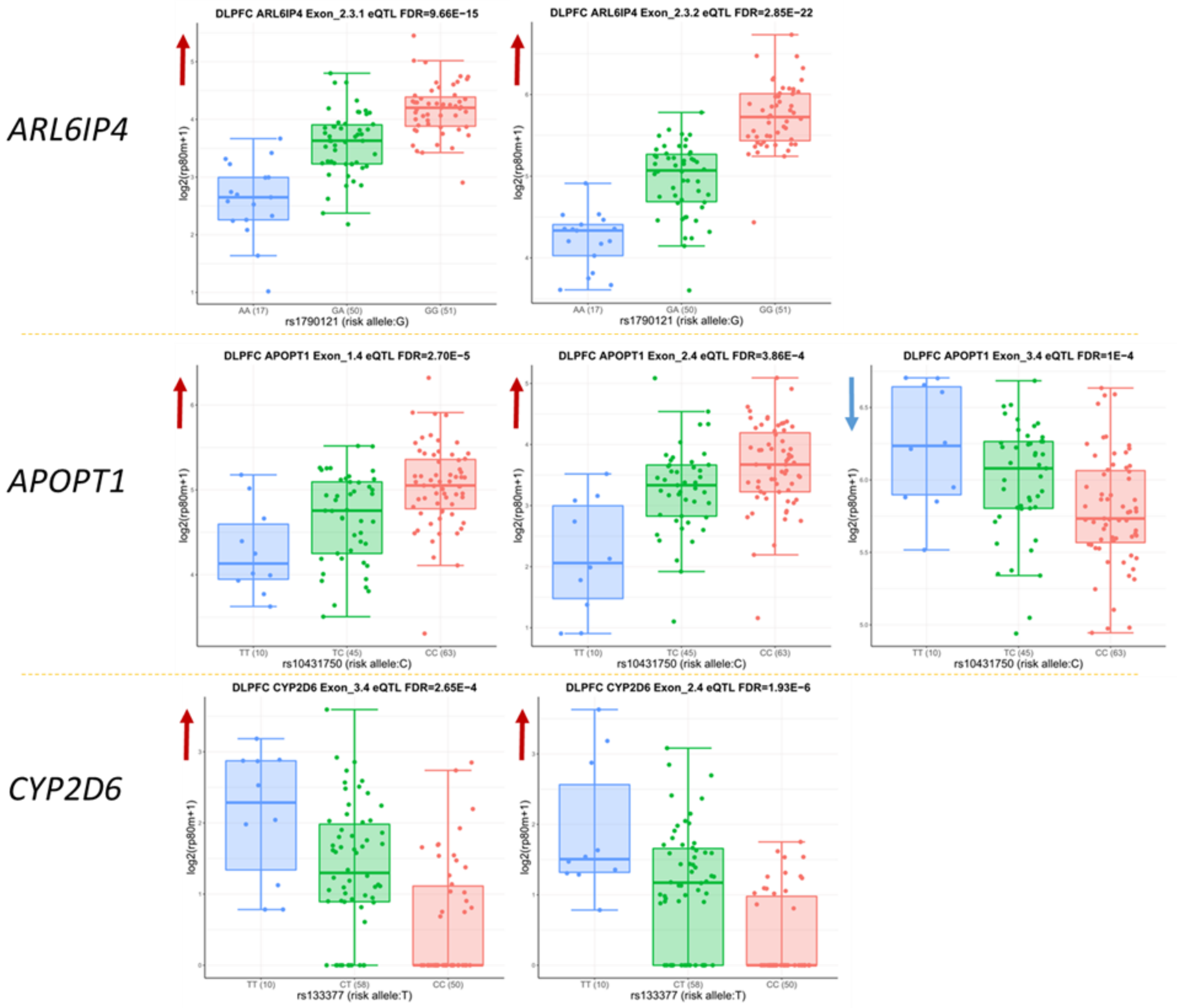

Supplementary Figure S6. Schizophrenia genetic risk effect on exon-exon junctions. Association of SNP-junction pairs: rs1790121 - ARL6IP4 Exon_2.3.1 (up) and Exon_2.3.2 (up); rs10431750 - APOPT1 Exon_1.4 (up) and Exon_2.4 (up) and Exon_3.4 (down); rs133377 - CYP2D6 Exon_34 (up) and Exon_24 (up). The junction abundance is from DLPFC. Red arrow indicates schizophrenia risk allele is associated with up regulation of junction, while blue arrow is associated with downregulation. SNX19 splicing junction Exon_8.10 was shown in our recent paper (Ma, et al., 2019).

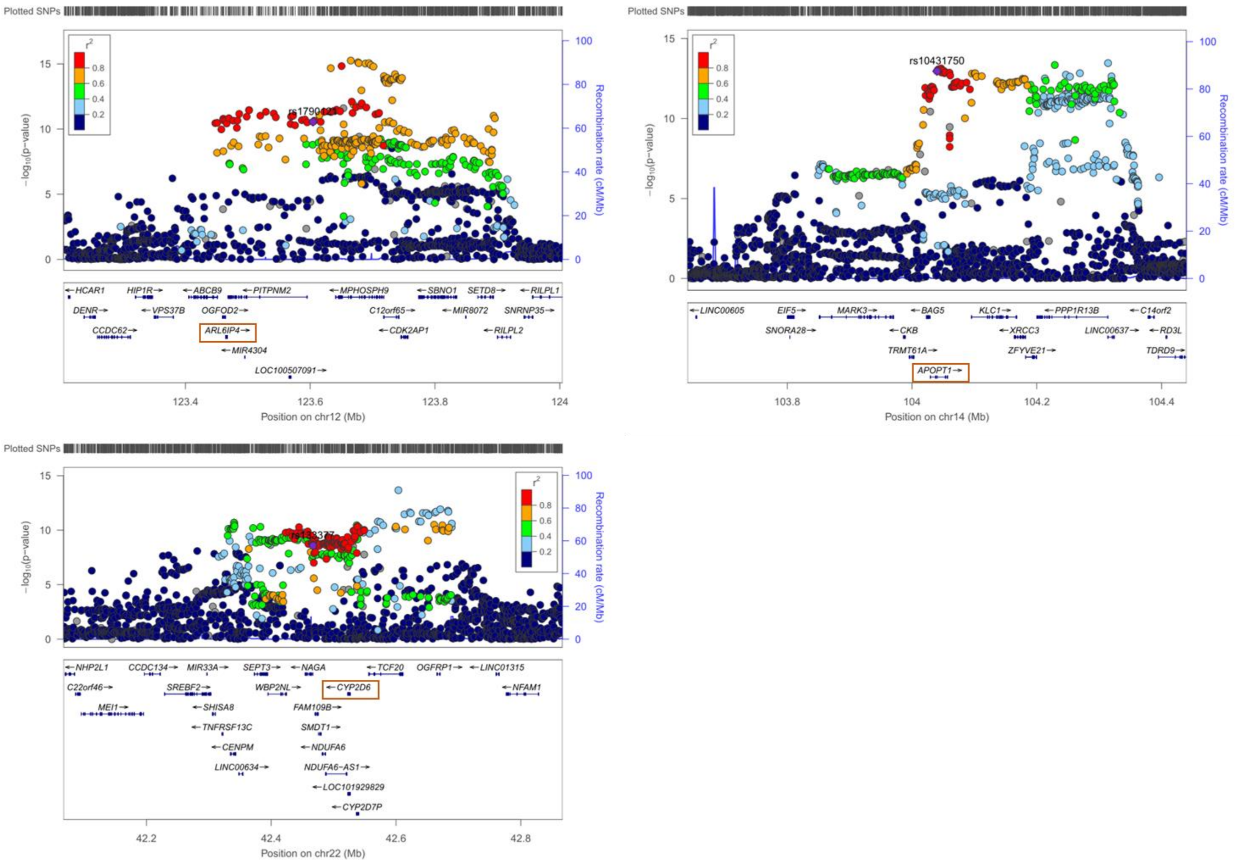

Supplementary Figure S7. Regional schizophrenia GWAS signature co-localized with our identified variants that influence splicing of APOPT1 (Exon_1.4 and Exon_2.4 and Exon_3.4), CYP2D6 (Exon_34 and Exon_24). Linkage disequilibrium is colored with respect to ARL6IP4 rs1790121, APOPT1 rs10431750, and CYP2D6 rs133377. See SNX19 in our prior work (Ma, et al., 2019).

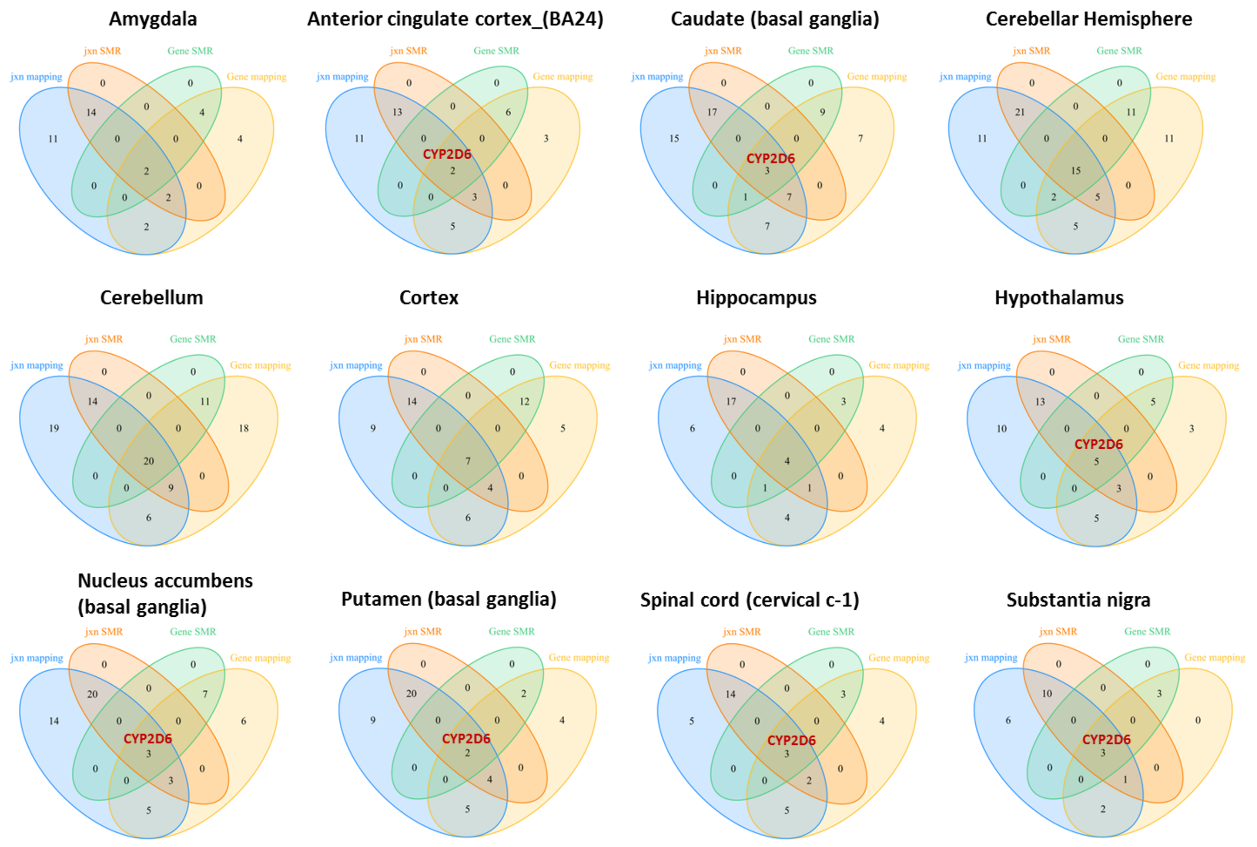

Supplementary Figure S8. Venn diagram of eGene and eJunction analysis across the 12 brain regions. Manhattan plots of dorsolateral prefrontal cortex (BA9) are shown in Figure 1.

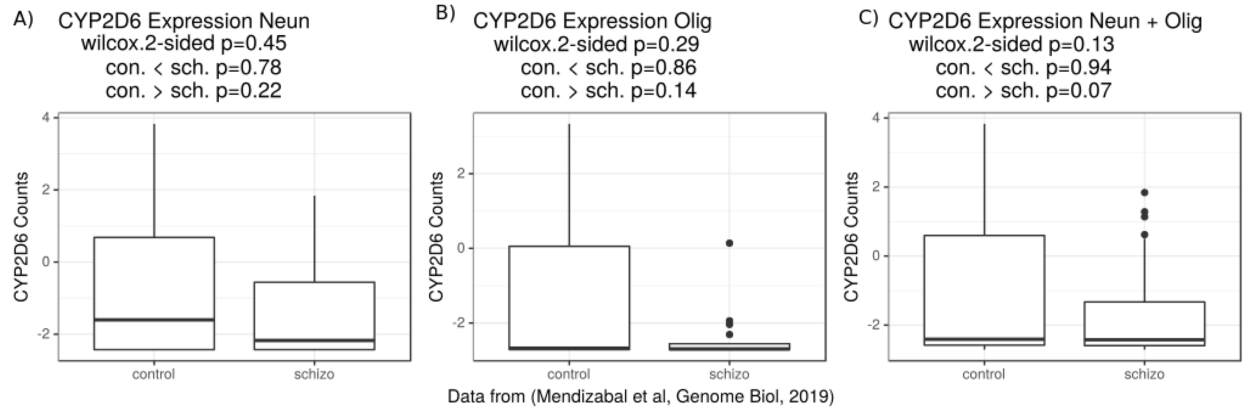

Supplementary Figure S9. CYP2D6 gene expression levels in schizophrenia (n=41) and control (n=50) oligodendrocytes and neurons (Mendizabal, et al., 2019). The Wilcoxon rank sum test (2-sided, expression in controls greater than expression in schizophrenia, expression in schizophrenia greater than controls) was performed in oligodendrocytes (A), neurons (B), and the combined sample set of oligodendrocytes and neurons (C).

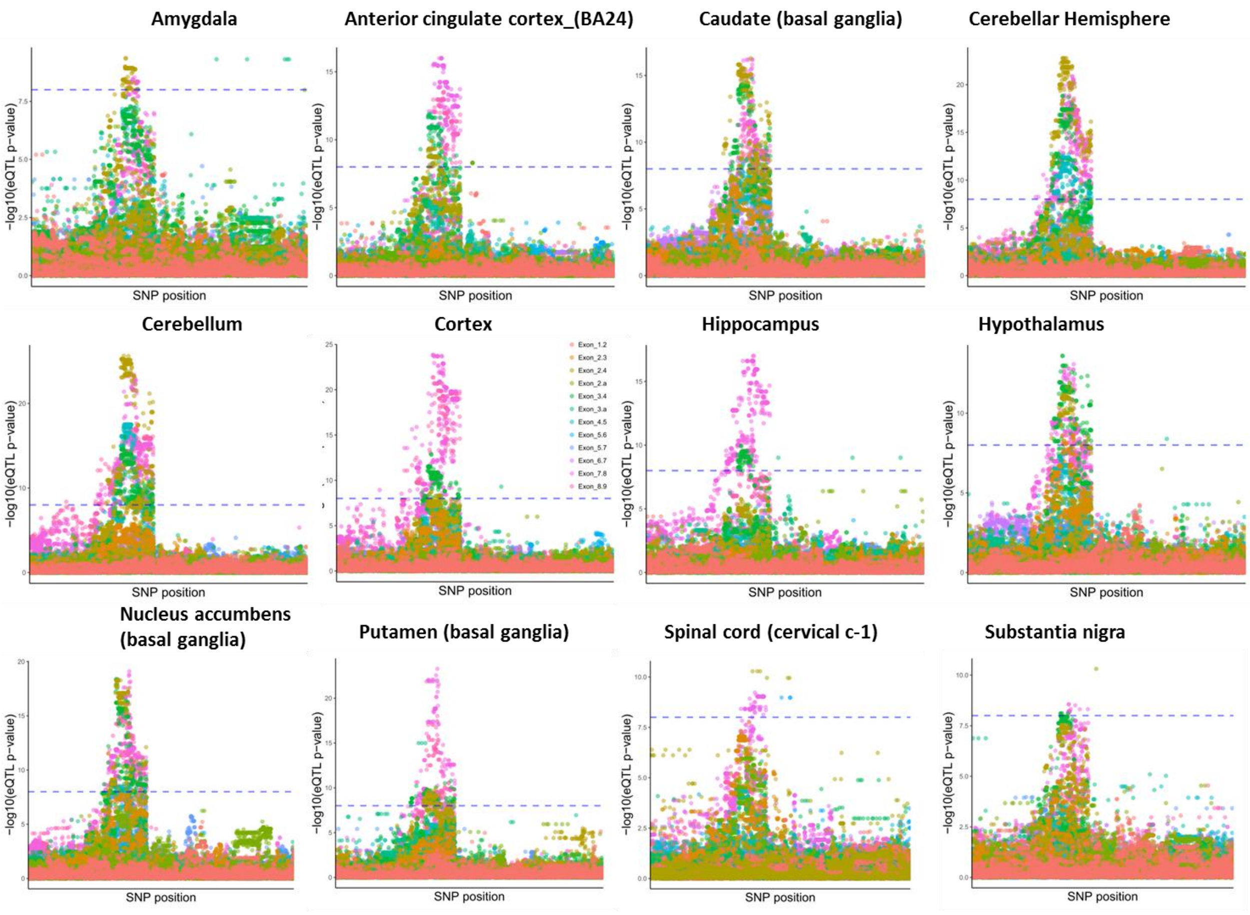

Supplementary Figure S10. Association of SNP-junction pairs (eJunction) of CYP2D6. See association results from DLPFC regions in Figure 4A.

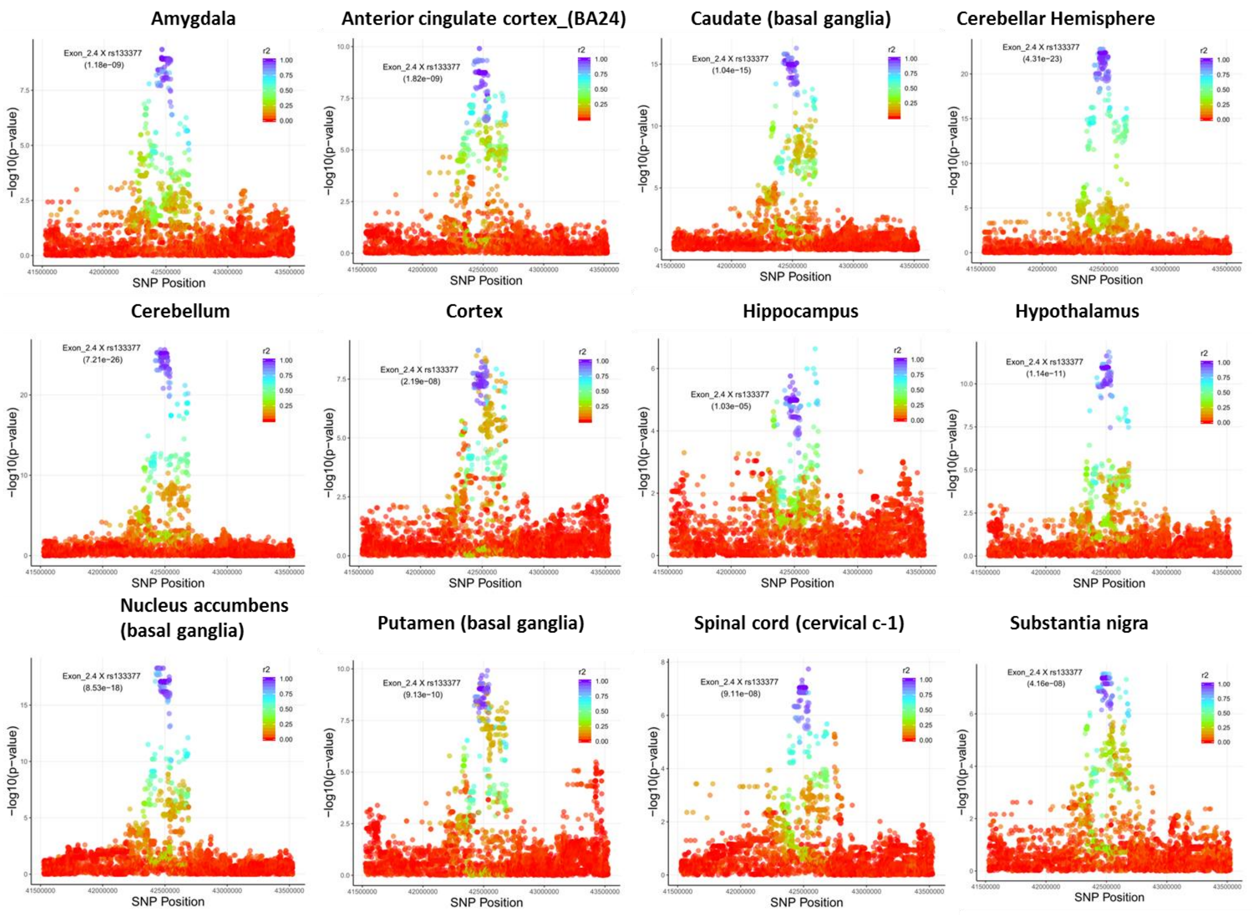

Supplementary Figure S11. Association of junctions Exon_2.4 with SNPs upstream and downstream of rs133377. r^2 was estimated using corresponding brain regions. See association results from DLPFC regions in Figure 4B.

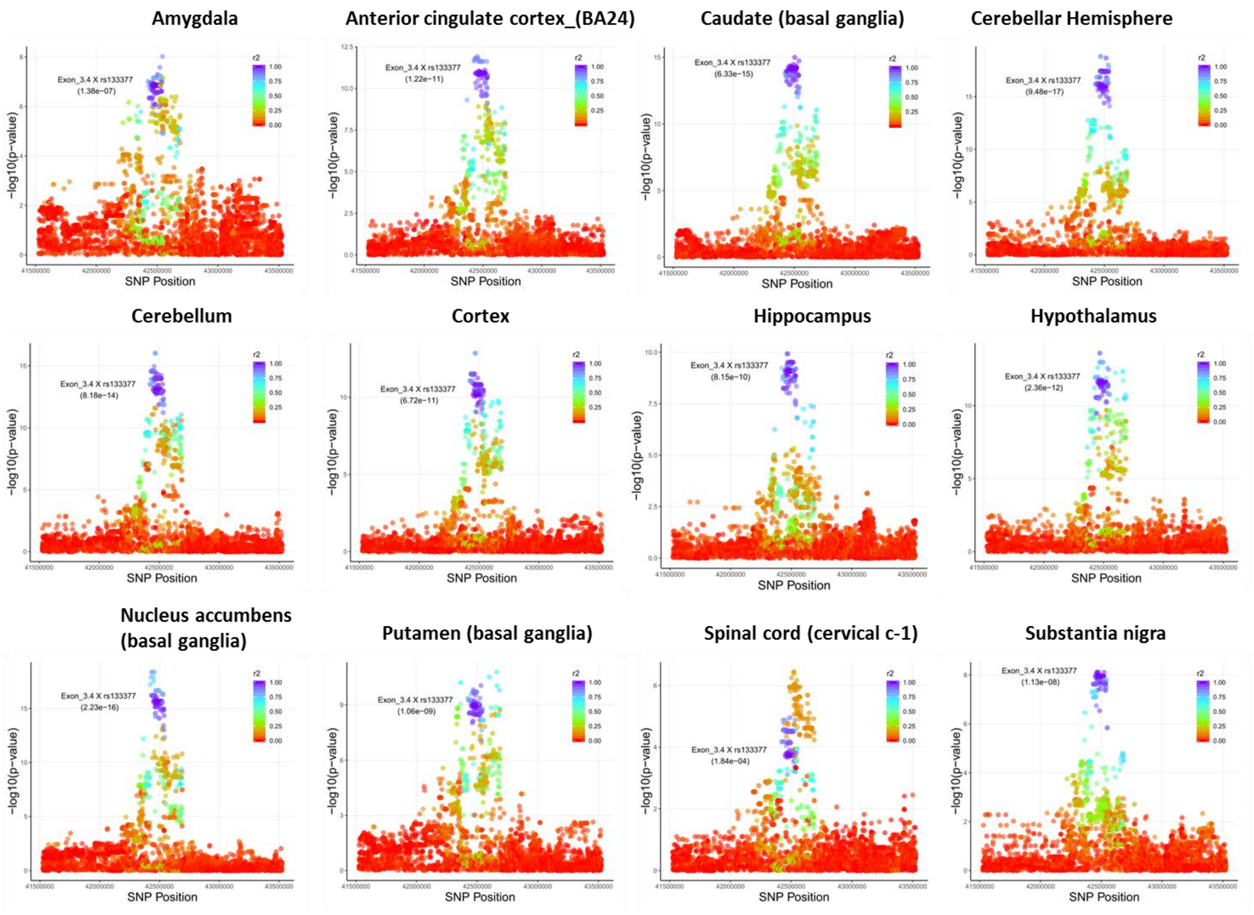

Supplementary Figure S12. Association of junctions Exon_3.4 with SNPs upstream and downstream of rs133377. r^2 was estimated using corresponding brain regions. See association results from DLPFC regions in Figure 4C.

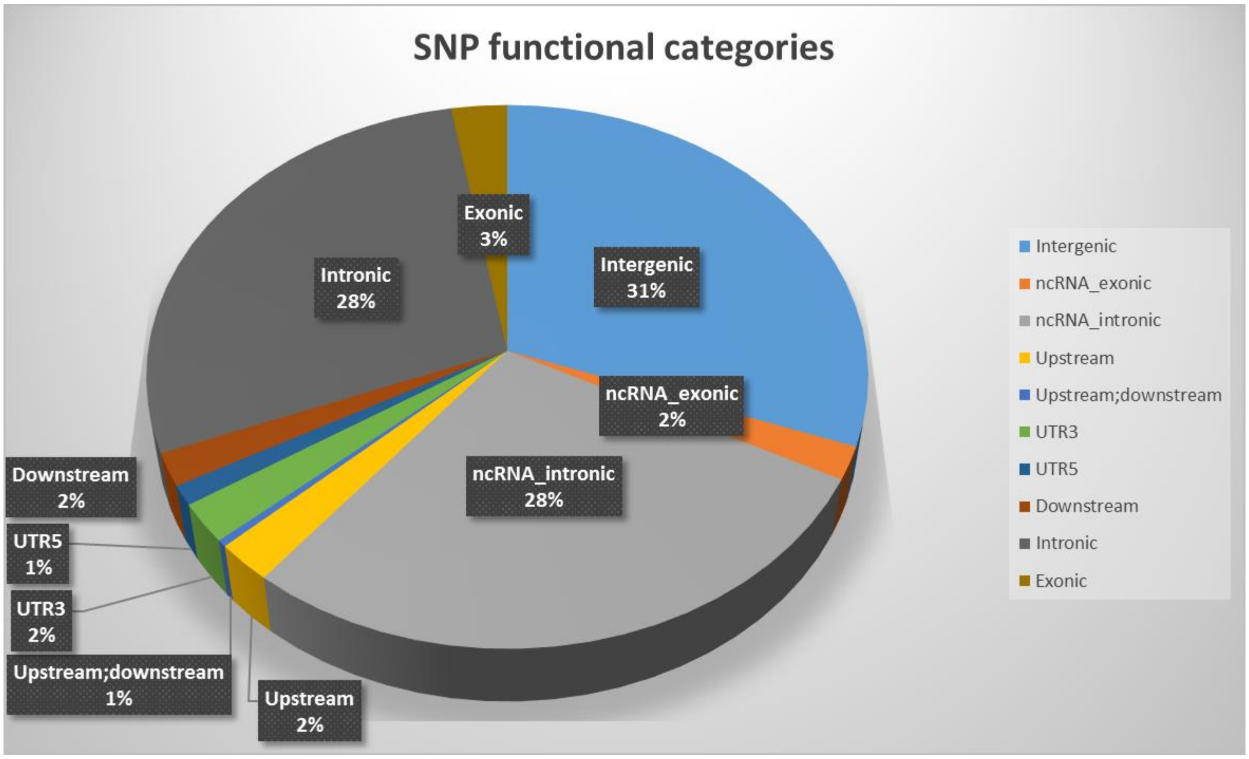

Supplementary Figure S13. Characterization of identified schizophrenia GWAS eJunction SNPs. Pie charts indicating proportions of SNPs annotated with each functional category (exonic, upstream, downstream, 3'-UTR, 5'-UTR, splicing, intronic and intergenic).

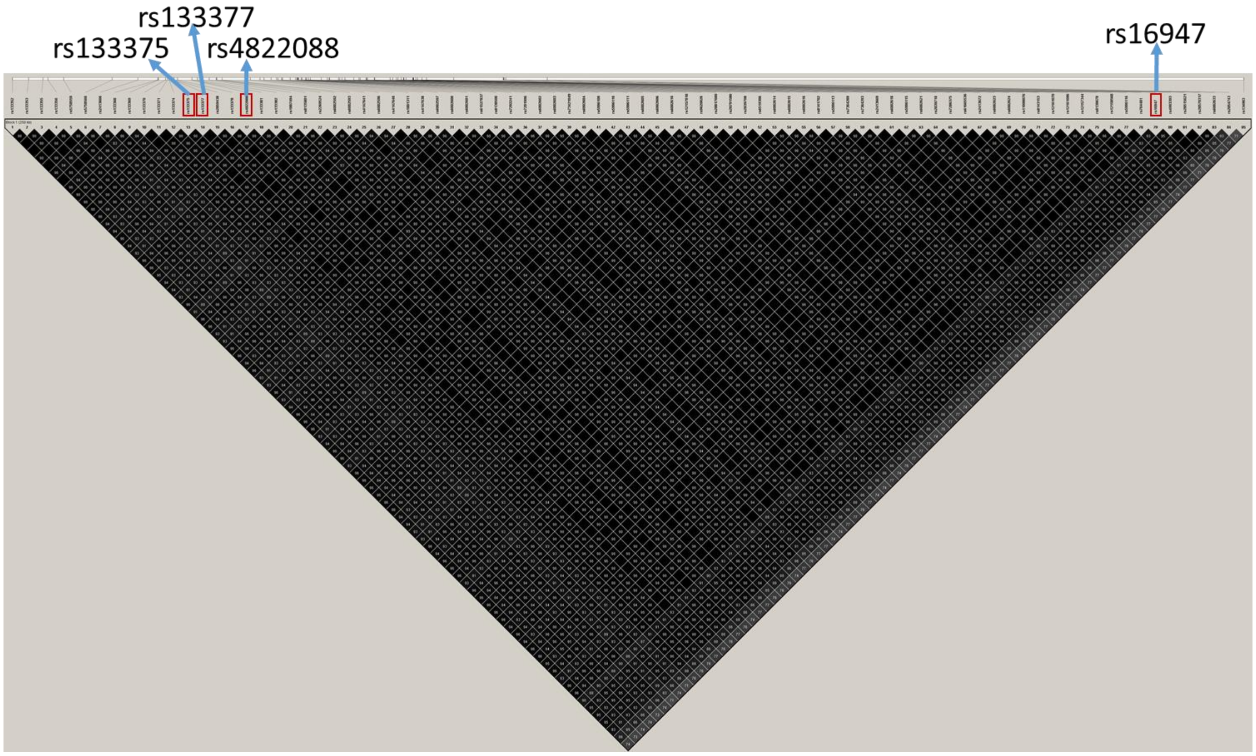

Supplementary Figure S14. Linkage disequilibrium (LD) plot of identified schizophrenia GWAS functional SNPs. r^2 was estimated using DLPFC data. See LD r^2 at Supplementary Table S6.

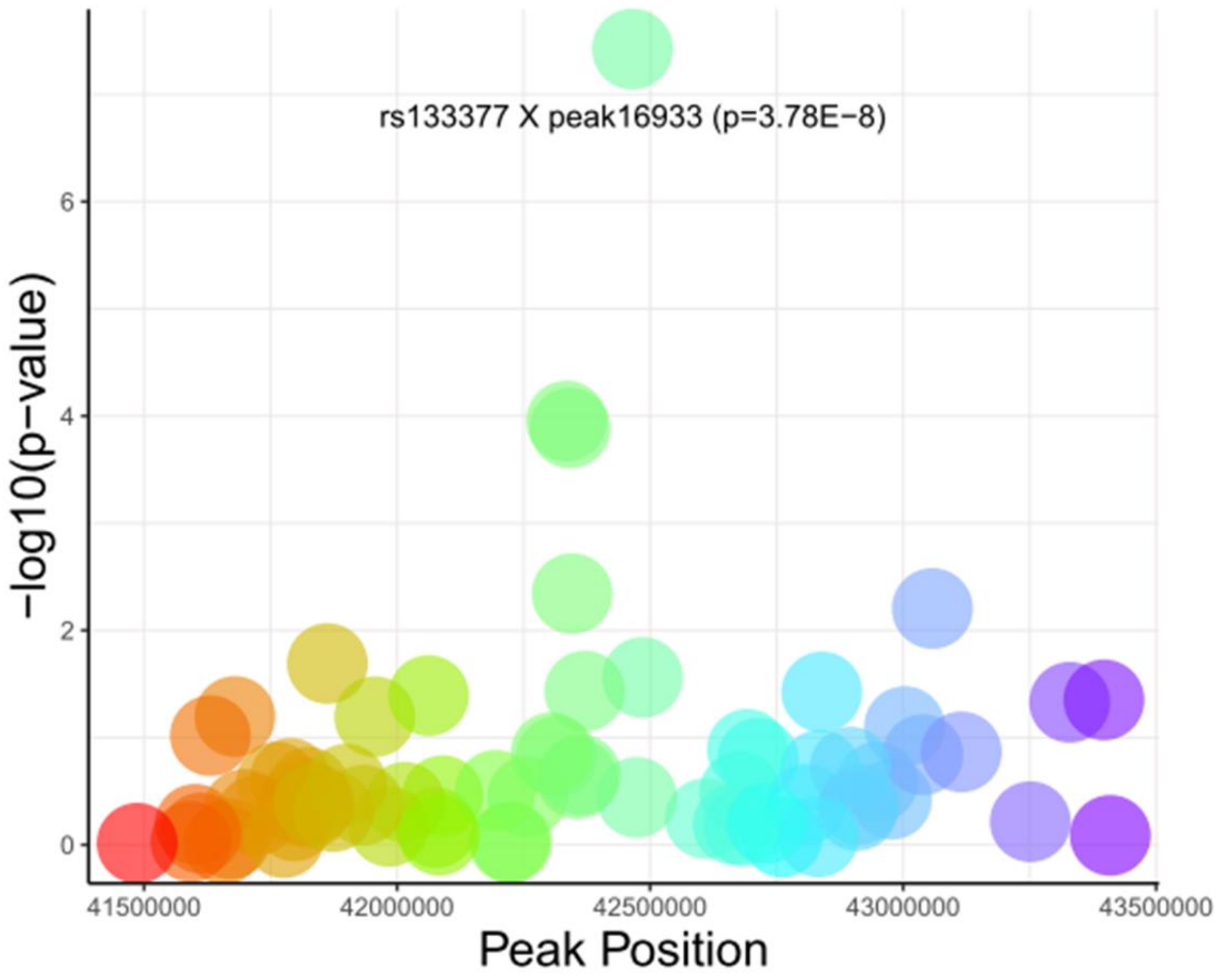

Supplementary Figure S15. Association of rs133377 (chr22:42466950) with 70 histone acetylation peaks in 2 MB region around CYP2D6.

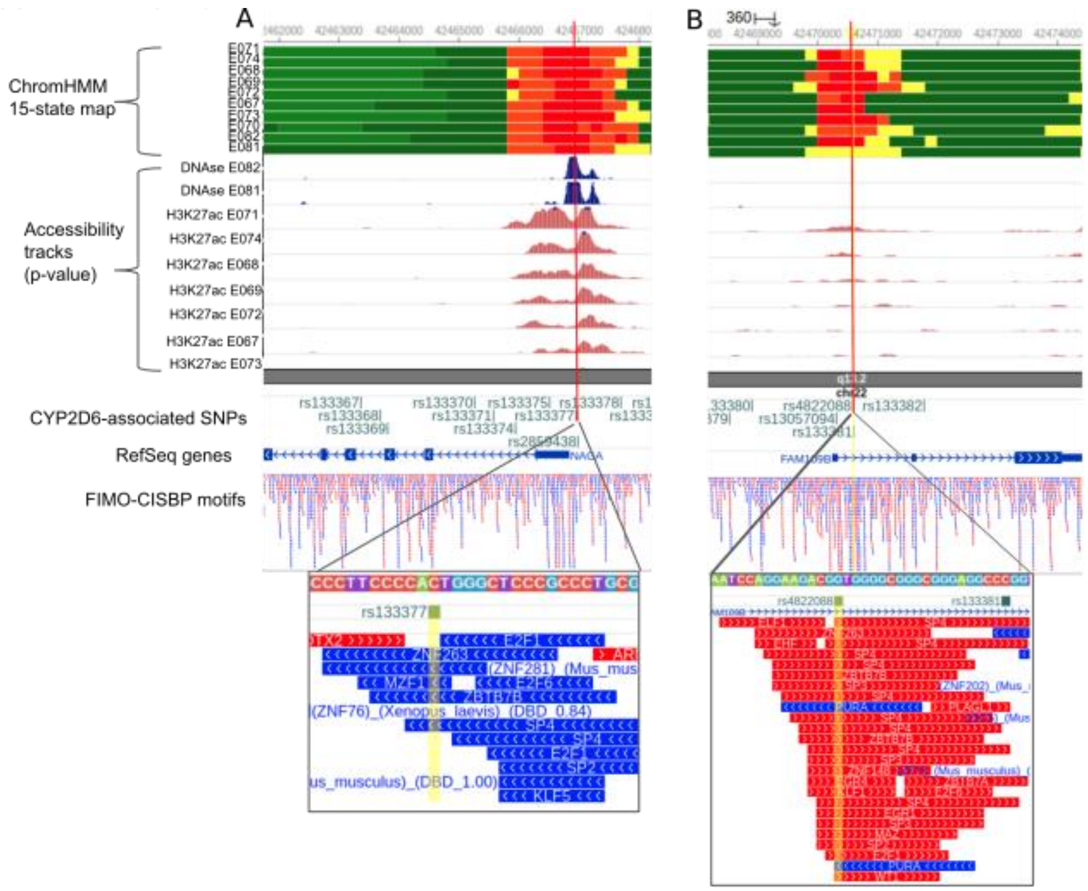

Supplementary Figure S16. Functional annotation for rs133377 (A) and rs4822088 (B). SNPs were overlapped with ChromHMM (15-state map) in Roadmap brain regions, DNAse data in Roadmap fetal brain samples (E081, E082 datasets), and H3K27ac Roadmap ChIP-seq datasets in the brain. Known TF binding sites in hg19 from FIMO and CISBP databases are overlapped with the SNP positions in the insets.

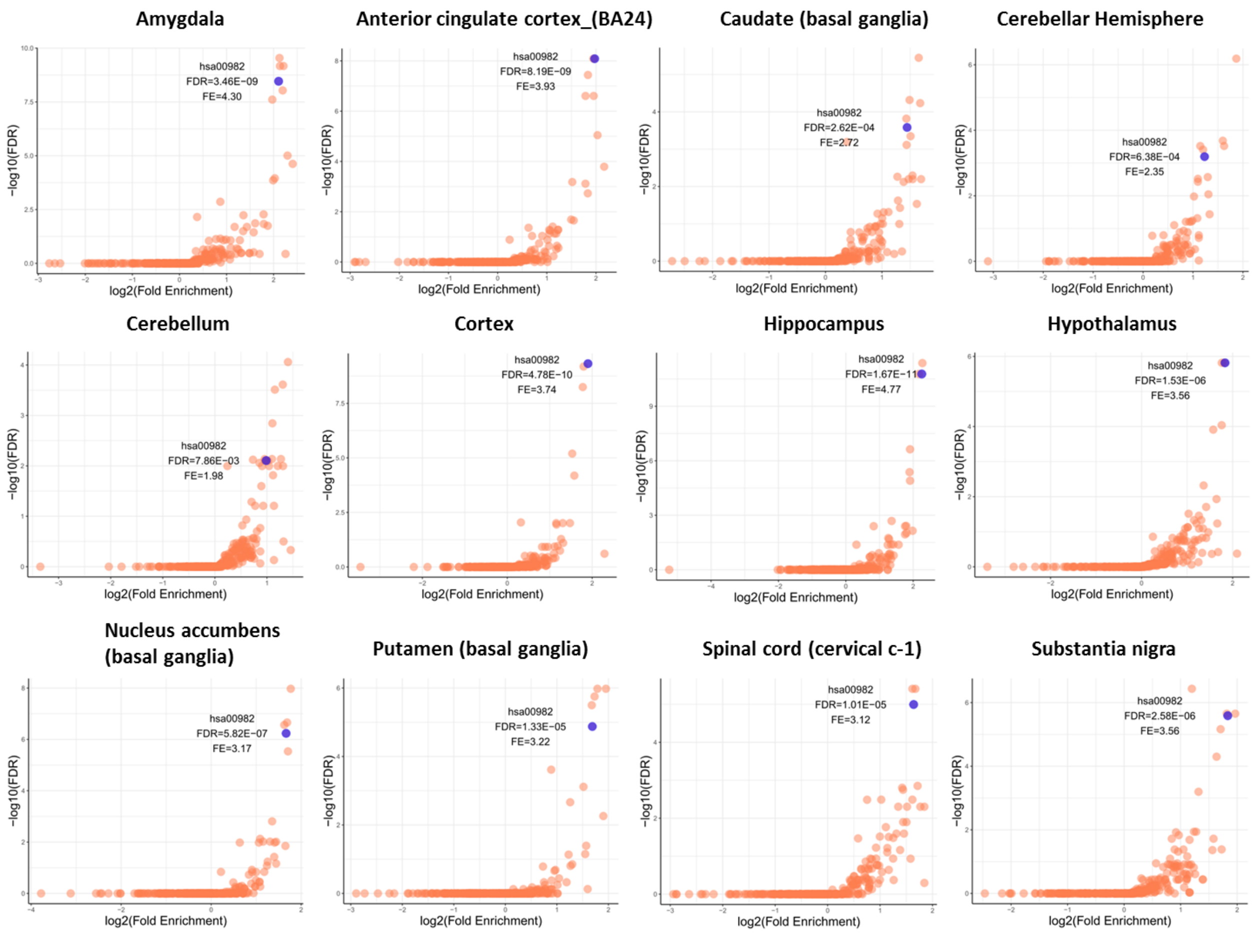

Supplementary Figure S17. Pathway analysis of overlapped significant genes of eJunctions and eGenes by WebGestalt across 12 brain regions.

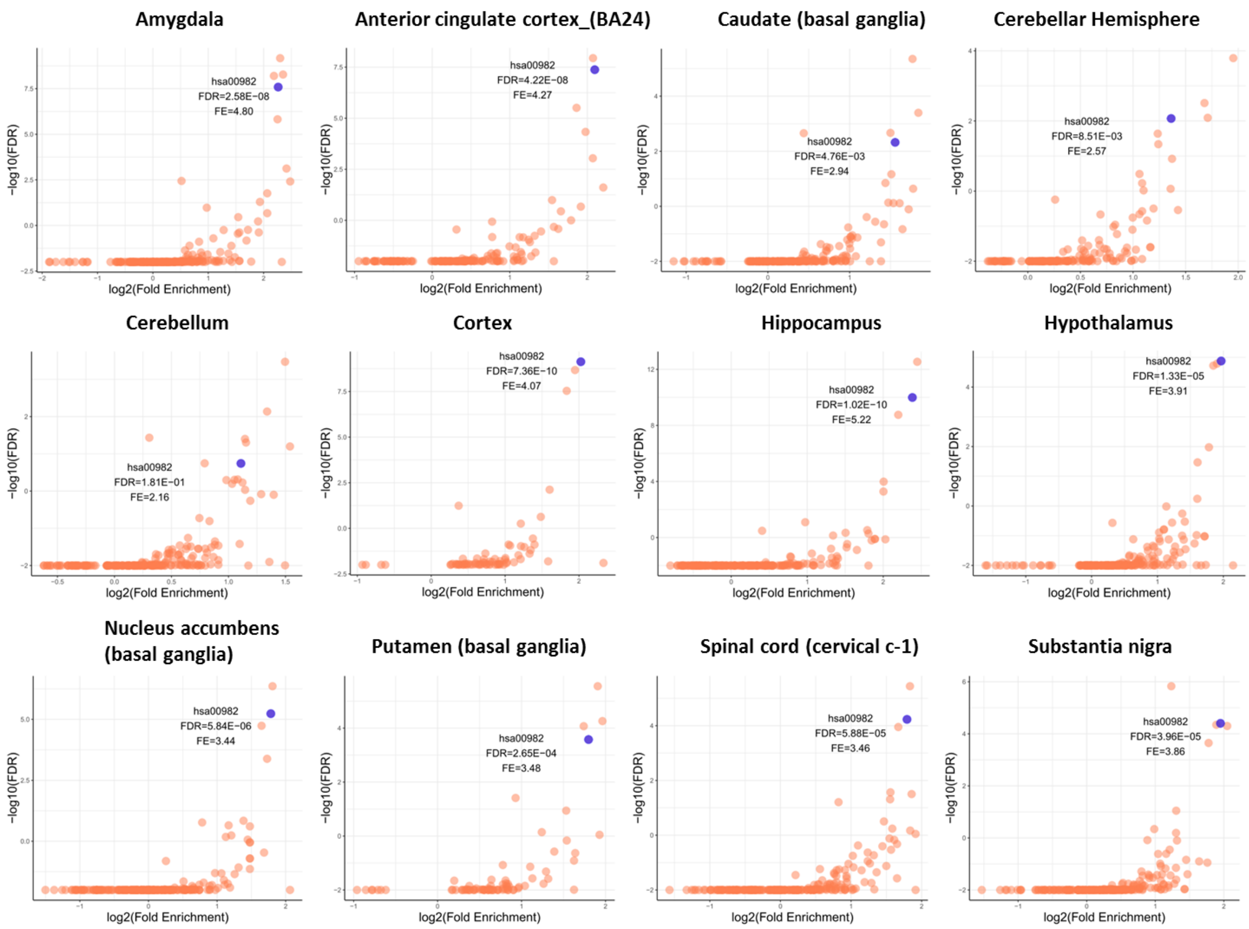

Supplementary Figure S18. Pathway analysis of overlapped significant genes of eJunctions and eGenes by DAVID across 12 brain regions.

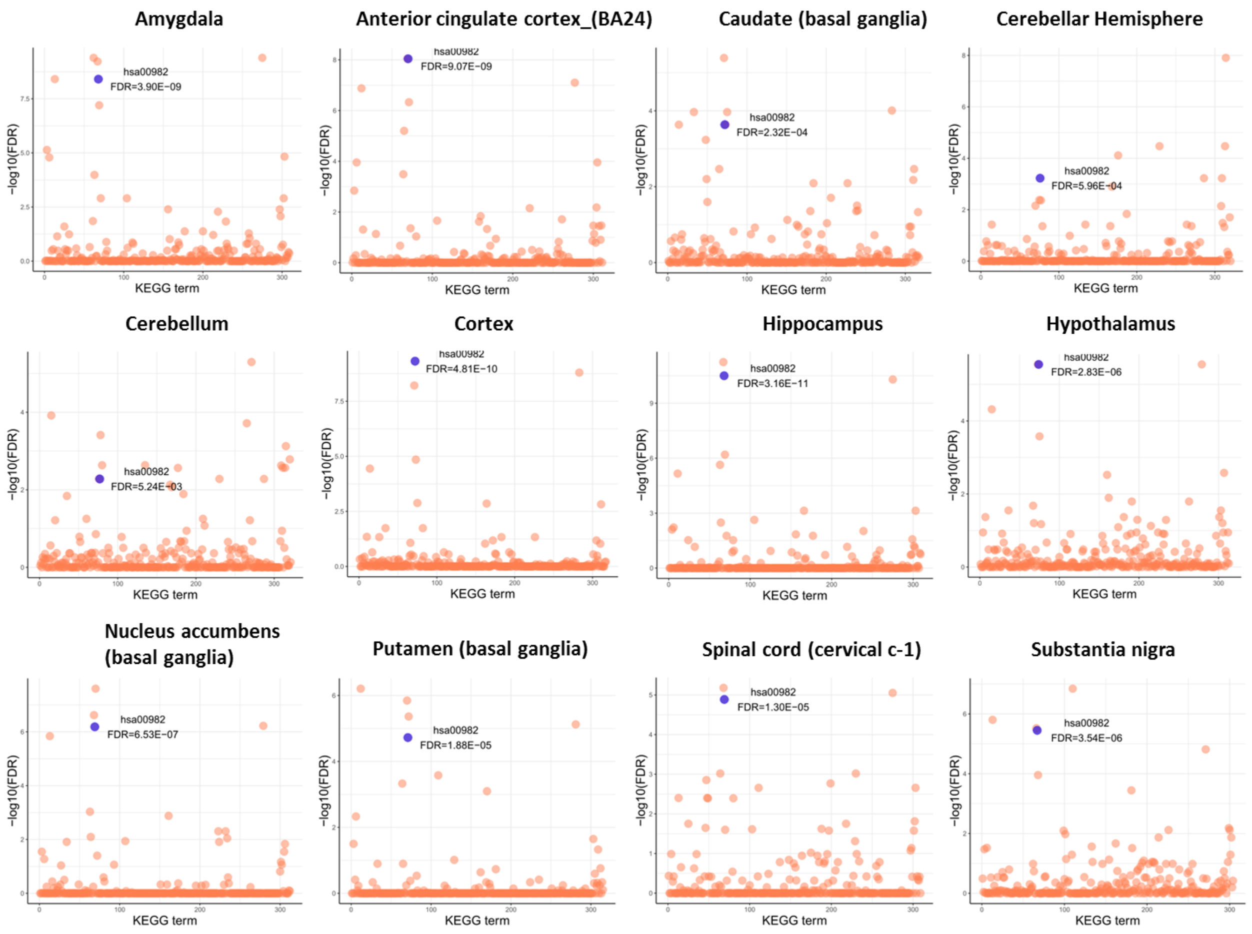

Supplementary Figure S19. Pathway analysis of overlapped significant genes of eJunctions and eGenes by gProfiler across 12 brain regions.

References

Chang CC, Chow CC, Tellier LC, et al. 2015. Second-generation PLINK: rising to the challenge of larger and richer datasets. Gigascience 4:7.

Ernst J, Kellis M. 2012. ChromHMM: automating chromatin-state discovery and characterization. Nat Methods 9(3):215-6.

Gene_Ontology_Consortium. 2015. Gene Ontology Consortium: going forward. Nucleic Acids Res 43(Database issue):D1049-56.

Grant CE, Bailey TL, Noble WS. 2011. FIMO: scanning for occurrences of a given motif. Bioinformatics 27(7):1017-8.

GTEx_Consortium, Laboratory DA, Coordinating Center -Analysis Working G, et al. 2017. Genetic effects on gene expression across human tissues. Nature 550(7675):204-213.

Gupta S, Stamatoyannopoulos JA, Bailey TL, et al. 2007. Quantifying similarity between motifs. Genome Biol 8(2):R24.

Harrow J, Frankish A, Gonzalez JM, et al. 2012. GENCODE: the reference human genome annotation for The ENCODE Project. Genome Res 22(9):1760-74.

Huang da W, Sherman BT, Lempicki RA. 2009. Systematic and integrative analysis of large gene lists using DAVID bioinformatics resources. Nat Protoc 4(1):44-57.

Jaffe AE, Straub RE, Shin JH, et al. 2018. Developmental and genetic regulation of the human cortex transcriptome illuminate schizophrenia pathogenesis. Nat Neurosci 21(8):1117-1125.

Kheradpour P, Kellis M. 2014. Systematic discovery and characterization of regulatory motifs in ENCODE TF binding experiments. Nucleic Acids Res 42(5):2976-87.

Ma L, Semick SA, Chen Q, et al. 2019. Schizophrenia risk variants influence multiple classes of transcripts of sorting nexin 19 (SNX19). Mol Psychiatry.

Mendizabal I, Berto S, Usui N, et al. 2019. Cell type-specific epigenetic links to schizophrenia risk in the brain. Genome Biol 20(1):135.

Ng B, White CC, Klein HU, et al. 2017. An xQTL map integrates the genetic architecture of the human brain's transcriptome and epigenome. Nat Neurosci 20(10):1418-1426.

Pardinas AF, Holmans P, Pocklington AJ, et al. 2018. Common schizophrenia alleles are enriched in mutation-intolerant genes and in regions under strong background selection. Nat Genet 50(3):381-389.

Price AL, Patterson NJ, Plenge RM, et al. 2006. Principal components analysis corrects for stratification in genome-wide association studies. Nat Genet 38(8):904-9.

Quinlan AR, Hall IM. 2010. BEDTools: a flexible suite of utilities for comparing genomic features. Bioinformatics 26(6):841-2.

Reimand J, Arak T, Vilo J. 2011. g:Profiler--a web server for functional interpretation of gene lists (2011 update). Nucleic Acids Res 39(Web Server issue):W307-15.

Shabalin AA. 2012. Matrix eQTL: ultra fast eQTL analysis via large matrix operations. Bioinformatics 28(10):1353-8.

Wang J, Vasaikar S, Shi Z, et al. 2017. WebGestalt 2017: a more comprehensive, powerful, flexible and interactive gene set enrichment analysis toolkit. Nucleic Acids Res 45(W1):W130-W137.

Wang K, Li M, Hakonarson H. 2010. ANNOVAR: functional annotation of genetic variants from high-throughput sequencing data. Nucleic Acids Res 38(16):e164.

Weirauch MT, Yang A, Albu M, et al. 2014. Determination and inference of eukaryotic transcription factor sequence specificity. Cell 158(6):1431-1443.

Zhu Z, Zhang F, Hu H, et al. 2016. Integration of summary data from GWAS and eQTL studies predicts complex trait gene targets. Nat Genet 48(5):481-7.
